# Simplified models to assess newborn gestational age in low-middle income countries: findings from a multicountry, prospective cohort study

**DOI:** 10.1136/bmjgh-2021-005688

**Published:** 2021-09-13

**Authors:** The Alliance for Maternal and Newborn Health Improvement (AMANHI) Gestational Age Study Group

**Keywords:** obstetrics, child health

## Abstract

**Introduction:**

Preterm birth is the leading cause of child mortality. This study aimed to develop and validate programmatically feasible and accurate approaches to estimate newborn gestational age (GA) in low resource settings.

**Methods:**

The WHO Alliance for Maternal and Newborn Health Improvement (AMANHI) study recruited pregnant women from population-based cohorts in five countries (Bangladesh, Ghana, Pakistan, Tanzania and Zambia). Women <20 weeks gestation by ultrasound-based dating were enrolled. Research staff assessed newborns for: (1) anthropometry, (2) neuromuscular/physical signs and (3) feeding maturity. Machine-learning techniques were used to construct ensemble models. Diagnostic accuracy was assessed by areas under the receiver operating curve (AUC) and Bland-Altman analysis.

**Results:**

7428 liveborn infants were included (n=536 preterm, <37 weeks). The Ballard examination was biased compared with ultrasound dating (mean difference: +9 days) with 95% limits of agreement (LOA) −15.3 to 33.6 days (precision ±24.5 days). A model including 10 newborn characteristics (birth weight, head circumference, chest circumference, foot length, breast bud diameter, breast development, plantar creases, skin texture, ankle dorsiflexion and infant sex) estimated GA with no bias, 95% LOA ±17.3 days and an AUC=0.88 for classifying the preterm infant. A model that included last menstrual period (LMP) with the 10 characteristics had 95% LOA ±15.7 days and high diagnostic accuracy (AUC 0.91). An alternative simpler model including birth weight and LMP had 95% LOA of ±16.7 and an AUC of 0.88.

**Conclusion:**

The best machine-learning model (10 neonatal characteristics and LMP) estimated GA within ±15.7 days of early ultrasound dating. Simpler models performed reasonably well with marginal increases in prediction error. These models hold promise for newborn GA estimation when ultrasound dating is unavailable.

**Key questions**

**What is already known?**

- In low-middle income countries, the gestational age (GA) dating of pregnancies is commonly inaccurate or unknown prenatally, and the GA of the baby is estimated after birth.
- Several clinical neonatal assessments exist to estimate the GA of the newborn, ranging from 4 to 23 physical and neurological signs.
- A recent systematic review and meta-analysis demonstrated that the most commonly used clinical assessment, the 10 sign Ballard score, systematically overestimated GA (3 days) and dated 95% of newborns within ±27 days (3.9 weeks) of ultrasound-based dating.
- The 21 sign Dubowitz examination dated 95% of newborns within ±18 days (2.6 weeks) of best obstetrical estimate.
- In general, clinical newborn assessments with fewer signs tend to be less accurate.

**Key questions**

**What are the new findings?**

- The WHO Alliance for Maternal and Newborn Health Improvement (AMANHI) GA study is a multicountry study that is one of the largest, well-dated, prospective, population-based pregnancy cohorts in low-middle income countries that included 7428 newborns from five countries in Africa and Asia.
- We aimed to develop the most precise model to predict newborn GA with the fewest clinical signs using machine learning techniques.
- In the most precise model that included 10 newborn characteristics (infant sex, five anthropometric measurements, three physical and one neurological sign) and last menstrual period (LMP), the model predicted GA within ±15.7 days (2.2 weeks) of early ultrasound and had high diagnostic accuracy for identifying preterm births.
- It correctly classified 91% of infants as preterm or not.
- In a simpler model including only two signs (birth weight and LMP), the prediction of GA was within ±16.7 days (2.4 weeks) of ultrasound and correctly classified 88% of infants as preterm or not.

**What do the new findings imply?**

- The AMANHI GA models may be used in clinical practice to more accurately identify high-risk, preterm infants in low-income settings and improve their access to special care.

## Introduction

Achieving meaningful declines in child mortality in the post-Millenium Development Goal era will require commitment and innovation to reduce mortality among babies born preterm (<37 weeks gestation). According to the latest WHO estimates, 14.8 million (10.6%) of newborns were born preterm worldwide in 2014.^1^ Preterm birth is the leading cause of under 5 child mortality, accounting for 15.9% of deaths globally.^2^ The risk of neonatal mortality among preterm infants is 6.8-fold higher than among infants born full-term.^3^

In low-middle income countries (LMICs), the lack of available or accurate data on the gestational age (GA) of a pregnancy, and thus misclassification of infant as preterm or not, is a critical barrier to providing adequate care for these vulnerable babies and estimating the global burden of preterm birth. Improvement in GA dating is a key priority to provide improved clinical care of mothers and babies, and to improve epidemiological data on the burden of disease. In the most recent estimates of preterm birth by WHO,^1^ the lack of quality GA data were a major limitation—with 91% of data used from high income or upper-middle income countries, and no GA data available from 76 out of 183 countries.^1^ GA in LMICs is commonly based on last menstrual period (LMP), and LMP recall is often unreliable,^4–6^ particularly in low-literacy populations. Ultrasonography coverage is low in sub-Saharan Africa and Asia. Moreover, presentation to antenatal care (ANC) for many women may be late in pregnancy in LMICs, when ultrasound is less accurate for dating. While increasing access to ultrasound is a priority for both maternal and newborn health, given the limitations and challenges to ANC access, there remains a critical need for new strategies to more accurately date newborns and identify preterm infants after birth.

For decades, the physical and neurological maturity of the newborn has been used to estimate the GA of the infant after delivery. In 1970, Dubowitz *et al* reported on a GA assessment including 21 external physical and neurological characteristics.^7^ In 1979, Ballard *et al* described a simplified score that required only 10 signs.^8^ Multiple scoring systems have been described in the literature ranging from 4 to 23 signs, including varying combinations and numbers of signs and measurements.^9^ In a recent systematic review, the Dubowitz examination was the most accurate method for the postnatal estimation of GA, dating 95% of newborns within ±2.6 weeks (18.2 days) of a best obstetrical estimate,^9^ while the Ballard examination was less precise (±3.8 weeks or 26.6 days). Generally, the fewer characteristics included in a scoring system, the more imprecise or inaccurate the estimates. However, feasibility is a critical consideration to implementation and scale in LMICs, where human resources are limited.

The WHO Alliance for Maternal and Neonatal Health Improvement (WHO AMANHI) study is a multicountry collaboration formed of investigators of maternal–newborn health studies from Bangladesh, Ghana, Pakistan, Tanzania and Zambia.^10^ To address the current limitations in GA dating, the main objective of the AMANHI GA study^10^ was to use novel techniques of machine learning to develop simple and programmatically feasible methods of estimating newborn GA following delivery in LMICs.

## Methods

### Study design and participants

The AMANHI GA study was conducted in prospective pregnancy cohorts in five sites—two in south Asia (Bangladesh (Sylhet), Pakistan (Karachi, Matiari)) and three in sub-Saharan Africa (Ghana (Brong Ahafo), Tanzania (Pemba) and Zambia (Southern Province)).^10^ Descriptions of the individual study sites and populations are detailed in online supplemental table 1. Uniform standard operating protocols and data tools were established and implemented across the sites.

10.1136/bmjgh-2021-005688.supp1 Supplementary data

**Table 1:** Newborn clinical signs assessed for in the Alliance for Maternal and Newborn Health Improvement Gestational Age study

Assessment	Signs
Neuromuscular signs	Posture
	Arm recoil
	Scarf sign
	Popliteal angle
	Heel-to-ear test
	Ankle dorsiflexion
Physical signs	Skin: Colour, texture, opacity and presence of lanugo
	Ear: Shape and recoil
	Breast: Nipple–areola development
	Male genitalia: Testes and scrotum
	Female genitalia: Labia and clitoris
	Foot: Plantar creases
Anthropometry	Head circumference (cm)
	Chest circumference (cm)
	Breast bud diameter (mm)
	Mid-upper arm circumference (cm)
	Foot length (mm)
	Infant length (cm)
	Symphysis–fundal height (cm)
	Weight (g)
Breast feeding Observation	Signs of attachment: more areola above infant’s top lip than below bottom lip; mouth wide open; lower lip everted; chin touching the breast
	Suckling behaviour: presence of deep, slow sucks with swallowing in between
	Duration the infant was able to stay attached to the breast continuously during the feed
	Longest continuous burst of suckling (number of sucks)
	Suck-to-swallow ratio

### Pregnancy identification

For all sites except Zambia, pregnancies were identified by population-based surveillance of women of reproductive age every 1–3 months. In Zambia, where over 96% of all pregnant women attend antenatal care clinics, the study recruited pregnant women from antenatal clinics. Women were considered eligible if they had a known LMP that suggested a GA of <20 weeks. Pregnant women were consented by field workers in the local language, and those consenting had an ultrasound scan for pregnancy dating.

### Ultrasonography

The ultrasound standard operations procedure (SOP) was developed by the AMANHI team with a maternal–fetal medicine specialist (BW). The SOP specified standardised procedures for measuring fetal biometric parameters transabdominally. Crown–rump length (CRL) was measured first. If CRL was >95 mm, both biparietal diameter (BPD) and femur length (FL) were additionally measured. At least two separate measurements were performed for each parameter. Average values were used for duplicate measures; median values were used for triplicate measures. If pregnancies were identified at <8 weeks, a repeat scan was scheduled 4 weeks later. Women with pregnancies enrolling >20 weeks were excluded from the GA study. To assign the gold standard GA, for scans with CRL between 15 and 95 mm, GA was assigned by the INTERGROWTH-21st formula.^11^ For participants who had CRL >95 mm, GA was assigned by averaging the GA determined by the BPD according to the formula of Hadlock *et al*^12^ and the GA determined by FL by Papageorghiou.^11^

### Neonatal assessment and anthropometrics

All pregnancies were followed until delivery and a neonatal assessment was conducted.

The neonatal examination included six neuromuscular signs of passive flexor tone or joint flexibility, and five physical signs from the original Ballard examination^8^ or Dubowitz examination^7^ (table 1). Neonatal anthropometry included infant weight, foot length (heel–halux), breast bud diameter, as well as head, chest and middle-upper arm circumference. Measurement scales and tools used to measure infant anthropometrics are detailed in online supplemental table 1. Signs of feeding maturity were adapted from WHO’s infant feeding assessment and the Nyqvist preterm feeding questionnaire.^13^

The newborn examination was conducted in most sites by non-clinician field workers (with at least 10 years formal education) and prior experience/training in maternal–newborn care (details in web online supplemental table 1). Infants were assessed within 72 hours of life; those infants deemed seriously ill were excluded.

#### Training and standardisation

WHO coordinated a centralised 3-day training of trainers in Sylhet, Bangladesh, to conduct training and standardisation of the neonatal assessment and anthropometrics (AM, ACL). After proficiency was established for each trainee with direct observation, a standardisation exercise was performed. Trainees were certified only after scoring all of the physical and neuromuscular signs within 1 point of the expert trainer on at least five newborns.

### Quality control

The WHO AMANHI coordinating team conducted central data review on a quarterly basis and conducted regular site visits to monitor field implementation and data collection. For ultrasound, a random selected 5% of images were sent for central review and feedback to an external maternal fetal medicine expert (BW). A standardised quality checklist of minimal acceptable quality standards for each biometric parameter was completed. For the newborn assessment, trained study coordinators independently conducted and/or directly observed a random 5% of neonatal assessments in the field to ensure maintenance of skill and quality.

### Statistical methods

For each subset of predictors considered, an ensemble model was constructed using the Super Learner algorithm^14^ as implemented in the R statistical software.^15^ The resulting model was a weighted average of multivariate adaptive regression splines,^16^ random forests,^17^ gradient boosting,^18^ support vector machines^19^ and multiple linear regression. Cross-validation was used both to determine optimal weights and to protect against overfitting of individual components of the ensemble and overly optimistic estimates of model performance.

It was not possible to fit ensemble models for all possible subsets of the 25 predictors available from the newborn assessments (ie, approximately 3.3 million subsets). A priori, we determined that 10 would be a maximum feasible number of signs to include in a newborn assessment for front-line health workers in LMICs. Predictors were prescreened using LASSO regularised regression^20^ and the designated ‘Top Ten’ model (model A) was identified by choosing a value for the regularisation parameter that resulted in 10 predictors. The initial screening of predictors retained various measures of anthropometry, along with scores of physical and neuromuscular development. Scores related to feeding maturity were excluded during the initial LASSO screening.

To compare the accuracy of the test methods and machine learning models for GA estimation, Bland-Altman plots were generated to summarise the agreement of GA as predicted by the test method versus gold standard (ultrasound) across the range of GA. Receiver operating curves were generated for each test method/model and areas under the receiver operating curve (AUC) calculated for the diagnostic accuracy of classifying infants <37 and <34 weeks. We assessed diagnostic accuracy by fixing sensitivity at a threshold of 80%, our a priori determined minimum sensitivity clinical threshold for a screening test for identifying preterm births. We also report diagnostic accuracy for fixed sensitivity of 85%, 90%, 95% and at the maximum Youden index, reflecting highest test accuracy.

### Sample size

In the AMANHI GA study, assuming that the simple machine learning models would detect a preterm prevalence of 10% with ±5% (absolute) precision and achieve 80% sensitivity and specificity to identify preterm infants in comparison to early pregnancy ultrasound dating, sample size was estimated to be 5740 pregnant women, an additional 2870 women were estimated to be required for validation of the machine learning model.^10^

### Patient and public involvement

Members of the public were not involved in the design or conduct of the study. During the formative/pilot phase of the study, family members of patients were involved in providing critical feedback on components of the neonatal assessment and certain procedures were modified based on their inputs. Specific examination components were eliminated (square window sign) and methods to calm the newborn were incorporated into study procedures.

## Results

### Study participant characteristics

**Figure 1:**
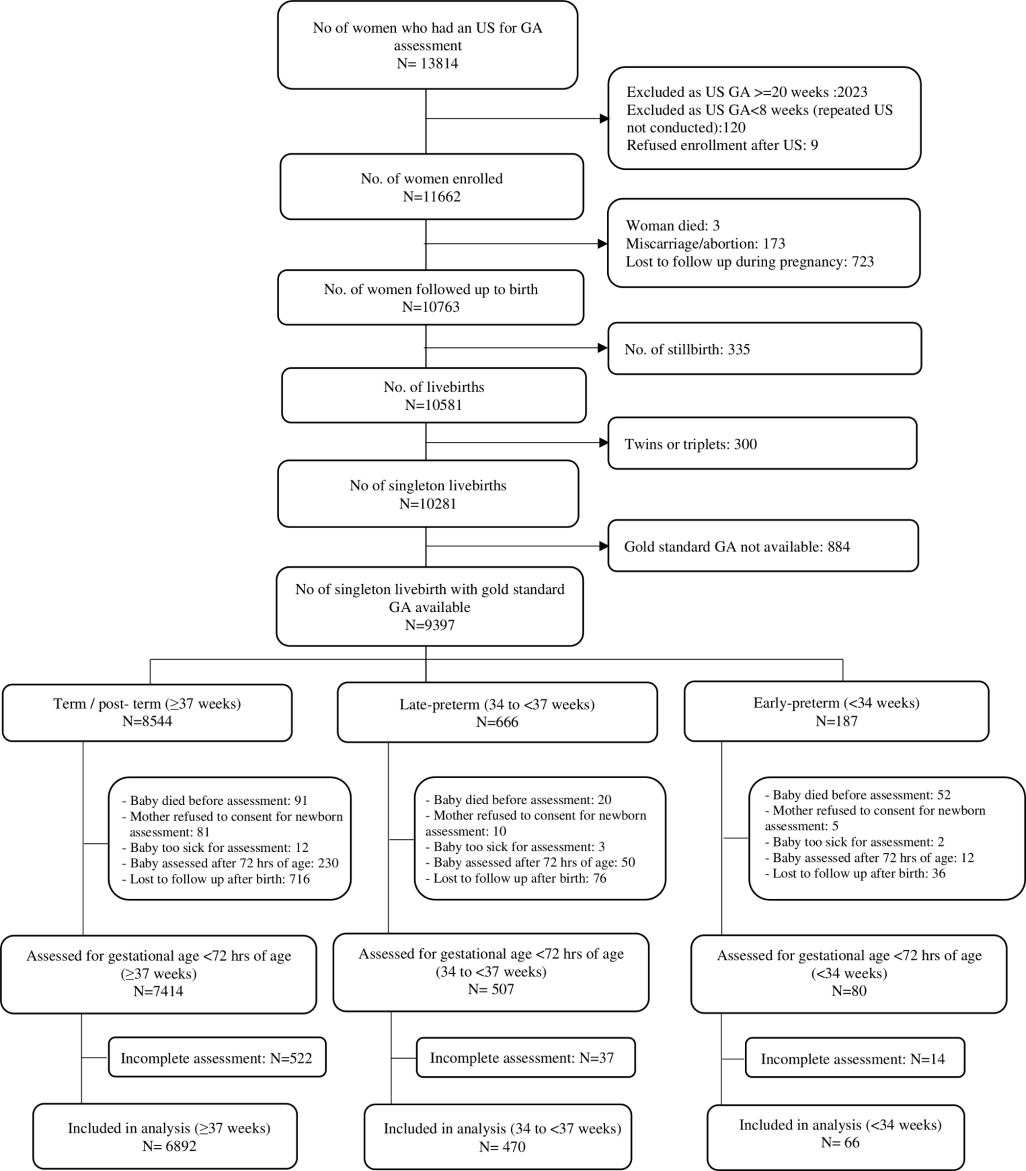
Flow chart of Alliance for Maternal and Newborn Health Improvement GA study participants. GA, gestational age; US, ultrasound.

From 1 January 2012 to 18 January 2017, a total of 11 662 pregnant women were enrolled, who had 10 581 live births (figure 1). Among these, there were 9397 singleton live births with early ultrasound dating available (n=8544 >37 weeks, 666 34–<37 weeks and 187 <34 weeks gestation). Of these 7414 (87%) term infants, 507 (76%) 34–<37 weeks infants, 80 (43%) <34 weeks infants were assessed at <72 hours of life. An important reason for the differential assessment was death prior to assessment (1% in term, 3% in late preterm (34–<37 weeks infants) and 28% in early preterm births (<34 weeks infants)). The majority (67%) of assessments were performed within the first 24 hours of life. Complete data on all examination components were found in 93% of assessed newborns and were included in the final analytic data set.

Basic characteristics of infants included in the analysis across study sites are shown in table 2. Women were somewhat younger in the Bangladesh and Zambia sites; and less educated in Pakistan and Ghana sites. Bangladesh and Pakistan sites had the lowest rates of facility births (44.1% and 63.1%, respectively). The prevalence of small for GA (SGA, <10% birth weight for GA and sex using the INTERGROWTH-21st standard)^21^ was higher in Bangladesh (42.1%), Pakistan (35.8%) and Ghana (32.7%).

**Table 2:** Characteristics of mothers–newborns included in the final analysis for Alliance for Maternal and Newborn Health Improvement Gestational Age study

	Bangladesh (N=1642)	Ghana (N=840)	Pakistan (N=2100)	Tanzania (N=2188)	Zambia (N=658)
Maternal characteristics					
Maternal age, n (%)*					
15–19	234 (18.6)	74 (8.8)	132 (7.6)	169 (7.8)	193 (29.6)
20–34	980 (77.9)	631 (75.4)	1439 (82.4)	1590 (72.9)	390 (59.7)
35+	44 (3.5)	132 (15.8)	175 (10)	422 (19.4)	70 (10.7)
Maternal parity, mean (SD)†	2.1 (1.5)	2.5 (1.8)	2.7 (2.1)	3.8 (2.4)	3.1 (2.1)
Maternal education, n (%)‡					
None	151 (9.6)	228 (27.3)	1048 (60)	289 (13.3)	12 (1.9)
1–6 years	698 (44.2)	523 (62.6)	278 (15.9)	748 (34.3)	53 (8.2)
7–12 years	721 (45.7)	69 (8.3)	408 (23.4)	1118 (51.3)	565 (87.6)
13+ years	9 (0.6)	16 (19)	12 (0.7)	26 (1.2)	15 (2.3)
Previous child death, n (%)§					
≥1 child death	224 (19.4)	152 (21.9)	132 (9.4)	274 (15.9)	45 (14.5)
Previous preterm birth, n (%)¶	12 (1.1)	11 (1.6)	34 (2.2)	28 (1.6)	17 (3.3)
Clean cooking fuel, n (%)**	16 (1)	87 (10.4)	1555 (89.1)	322 (14.8)	42 (6.5)
Improved latrine facility, n (%)††	1570 (96.5)	663 (79.3)	1638 (96.4)	1605 (73.6)	585 (90.1)
Pre-eclampsia or eclampsia during pregnancy, n (%)‡‡	2 (0.2)	7 (0.8)	9 (0.6)	87 (4)	0 (0)
Birth characteristics					
Health facility delivery, n (%)§§	699 (44.1)	664 (79.1)	1326 (63.1)	2135 (99.9)	602 (97.3)
Skilled birth attendant, n (%)¶¶	672 (42.4)	652 (77.7)	1427 (68)	1570 (73.5)	554 (91.7)
Type of delivery, n (%)***					
Normal vaginal delivery	1506 (94.7)	739 (88.1)	1786 (88.9)	2064 (96.8)	612 (96.8)
Assisted vaginal delivery	26 (1.6)	7 (0.8)	30 (2)	7 (0.3)	0 (0)
C-section	58 (3.7)	93 (11.1)	202 (10)	62 (2.9)	13 (3)
Low birth weight, n (%)					
(<2500 g)	431 (26.3)	98 (11.7)	493 (23.5)	101 (4.6)	43 (6.5)
Small for gestational age, n (%)	691 (42.1)	275 (32.7)	751 (35.8)	206 (9.4)	119 (18.1)

Missing data are listed as (n=Bangladesh, Ghana, Pakistan, Tanzania, Zambia).

*Missing maternal age data (n=384, 3, 354, 7, 5).

†Missing parity data (n=48, 3, 163, 44, 212).

‡Missing maternal education data: (n=63, 4, 354, 7, 13).

§Missing previous child death data: (n=48, 3, 311, 109, 212).

¶Missing previous preterm birth data: (n=55, 3, 175, 109,79).

**Missing clean cooking fuel data: (n=51, 4, 354, 7, 10).

††Missing improved latrine facility data: (n=15, 4, 354, 7, 9).

‡‡Missing pre-eclampsia/eclampsia data: (n=388, 0, 664, 14, 5).

§§Missing health facility delivery data: (n=58, 1, 0, 51, 39).

¶¶Missing skilled birth attendant data: (n=58, 1, 0, 51, 54).

***Missing type of delivery data: (n=52, 1, 70, 51, 221).

### Diagnostic accuracy of existing clinical methods to determine GA

**Figure 2:**
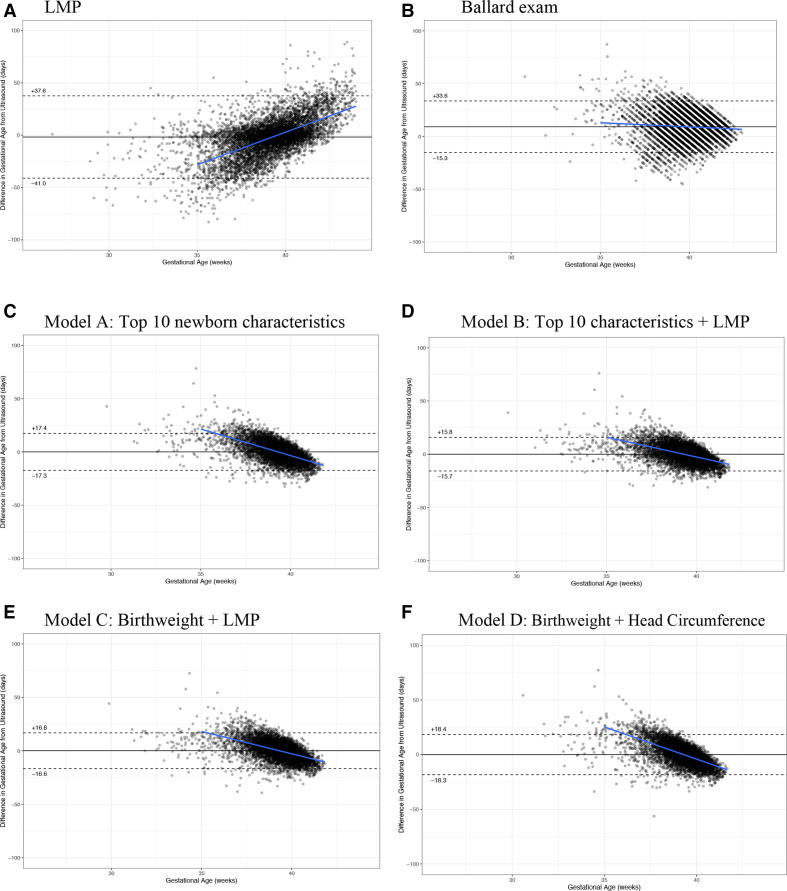
(A–F) Bland-Altman plots (with bias and trendline). (A) LMP, (B) Ballard examination, (C) model A: top 10 newborn characteristics, (D) model B: top 10 characteristics+LMP, (E) model C: birth weight+LMP and (F) model D: birth weight+head circumference. LMP, last menstrual period.

We determined the accuracy of the LMP and Ballard examination dating compared with early ultrasound dating (tables 3 and 4, figure 2A, B).

**Table 3:** Agreement between early ultrasound dating versus gestational age (GA) determined by last menstrual period (LMP), Ballard Score and AMANHI machine learning models (A, B, C, D)

GA determined by:	N	Mean difference in days (95% CI) (GA test method—GA ultrasound)	Bland-Altman 95% limits of agreement (in days)	Precision of predicted GA (in days)*
**LMP† (<37 weeks**)	7428	−1.69 (−2.15 to –1.24)	(−41.0 to 37.6)	±39.3
**Original Ballard Score‡**	7428	9.16 (8.87 to 9.44)	(−15.3 to 33.6)	±24.5
**AMANHI model A** Newborn 10-characteristics § (including birth weight)	7428	0.03 (−0.17 to 0.23)	(−17.3 to 17.4)	±17.3
**AMANHI model B** Newborn 10-characteristics§+LMP†	7428	0.03 (−0.15 to 0.22)	(−15.7 to 15.8)	±15.7
**AMANHI model C** Birth weight+LMP†	7428	0.07 (−0.13 to 0.26)	(−16.6 to 16.8)	±16.7
**AMANHI model D** Birth weight+head circumference	7428	0.06 (−0.15 to 0.28)	(−18.3 to 18.4)	±18.4

*Interpretation: Refers to the precision of the predicted GA values estimated by the test method bias (95% CI of the individual differences) around the mean difference.

†LMP: In this study LMP was collected from maternal recall at <20 weeks gestation in all sites as part of prospective research studies.

‡Ballard Score: GA was calculated from the Ballard signs as described in Ballard *et al*^29^ using the formula GA=((2×score)+120))/5.

§AMANHI 10-characteristics: Birth weight, head circumference, chest circumference, foot length, breast bud diameter, breast development, foot surface (plantar creases), skin texture, ankle dorsiflexion, infant sex.

AMANHI, Alliance for Maternal and Newborn Health Improvement.

**Table 4:** Diagnostic accuracy of Ballard, LMP and AMANHI models for identification of newborns <37 and <34 weeks (gold standard dated by early pregnancy ultrasound)

Model	Area under the curve	Cut-off selection	Sensitivity	Specificity	Positive predictive value	Negative predictive value	Positive likelihood ratio	Negative likelihood ratio
Classify <37 weeks*								
LMP (<37 weeks)†	0.81	LMP <37 weeks cut-off	0.69	0.81	0.22	0.97	3.72	0.38
LMP	0.81	80% sensitivity	0.80	0.71	0.18	0.98	2.78	0.28
Ballard exam‡	0.74	BS cut-off <37 weeks	0.09	0.98	0.25	0.93	4.29	0.93
Ballard exam	0.74	80% sensitivity	0.80	0.52	0.12	0.97	1.68	0.38
Model A (10-characteristics)	0.88	80% sensitivity	0.80	0.80	0.23	0.98	3.93	0.25
Model B (10-characteristics+LMP)	0.91	80% sensitivity	0.80	0.87	0.32	0.98	5.96	0.23
Model C (BW+LMP)	0.88	80% sensitivity	0.80	0.80	0.23	0.98	3.95	0.25
Model D (BW+HC)	0.84	80% sensitivity	0.80	0.72	0.18	0.98	2.85	0.28
Classify <34 weeks§								
LMP¶	0.94	LMP <34 weeks cut-off	0.61	0.95	0.10	1.00	12.93	0.41
LMP	0.94	80% sensitivity	0.80	0.90	0.07	1.00	8.05	0.22
Ballard**	0.89	BS cut-off <34 weeks	0.03	1.00	0.40	0.99	74.36	0.97
Ballard	0.89	80% sensitivity	0.80	0.81	0.04	1.00	4.13	0.25
Model A (10-characteristics)	0.94	80% sensitivity	0.80	0.96	0.16	1.00	21.38	0.21
Model B (10- characteristics+LMP)	0.96	80% sensitivity	0.80	0.98	0.31	1.00	50.39	0.20
Model C (BW+LMP)	0.96	80% sensitivity	0.80	0.98	0.23	1.00	34.24	0.20
Model D (BW+HC)	0.93	80% sensitivity	0.80	0.93	0.10	1.00	12.23	0.21

Diagnostic accuracy for different cut-offs with preset sensitivity of 85%, 90% and 95% are shown in online supplemental table 2.

*In the cohort there were 536 infants that were classified as preterm <37 weeks, and 6892 classified as ≥37 weeks.

†LMP <37 weeks was classified as preterm, and LMP ≥37 weeks classified as full term.

‡GA as determined by Ballard score was classified as preterm if GA <37 weeks using the equation GA=((2×score)+120))/5.

§In the cohort there were 66 infants that were classified as preterm <34 weeks, and 7362 classified as ≥34 weeks.

¶Threshold of GA determined by LMP <34 weeks.

**Original Ballard score equation (as per footnote 3) classification.

AMANHI, Alliance for Maternal and Newborn Health Improvement; BS, Ballard Score; BW, birth weight; GA, gestational age; HC, head circumference; LMP, last menstrual period.

#### Last menstrual period

The average bias of LMP was 1.7 days underestimation compared with ultrasound (table 3), with a trend of underestimation of GA at lower ranges of GA and overestimation at higher GA (figure 2A). The 95% limits of agreement (LOA) were −41.0 to 37.6 days. Using an LMP cut-off of <37 weeks, the sensitivity and specificity of identifying preterm birth based on ultrasound was 69% and 81%, respectively (table 4).

#### Clinical Ballard examination

The Ballard exam systematically overestimated GA by 9.2 days (95% CI 8.9 to 9.4), and had a 95% LOA (−15.3, 33.6 days) compared to early ultrasound dating. This bias towards GA overestimation was consistent in the country specific analysis (online supplemental figures 2–6). Using the standard Ballard calculation of gestational age, the sensitivity of the Ballard exam to identify preterm and early preterm infants was very low (9% and 3%, respectively) (table 4).

**Figure 3:**
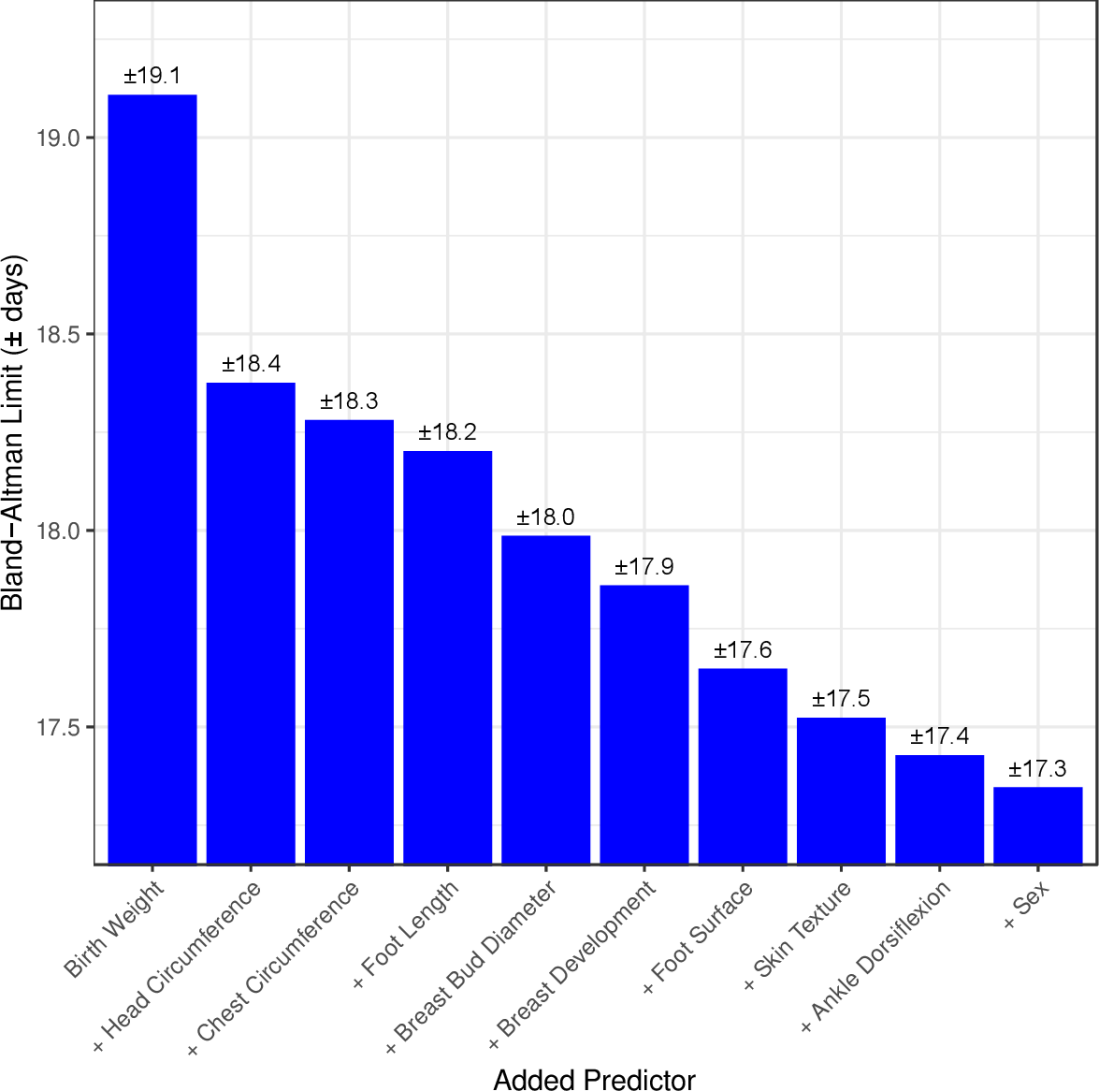
Ranking of 10 top predictors included in the machine learning model*. *The limits of agreement on Y axis indicate that 95% of estimated values of gestational age (GA) estimated by the machine learning model including the predictor are within ±y value days of the gold standard ultrasound estimated GA (machine learning models have zero mean bias). **Each predictor listed is cumulative, that is, in addition to the aforementioned predictors. (ie, machine learning model with birth weight AND head circumference predict GA within ±18.4 days of early ultrasound GA).

**Figure 4:**
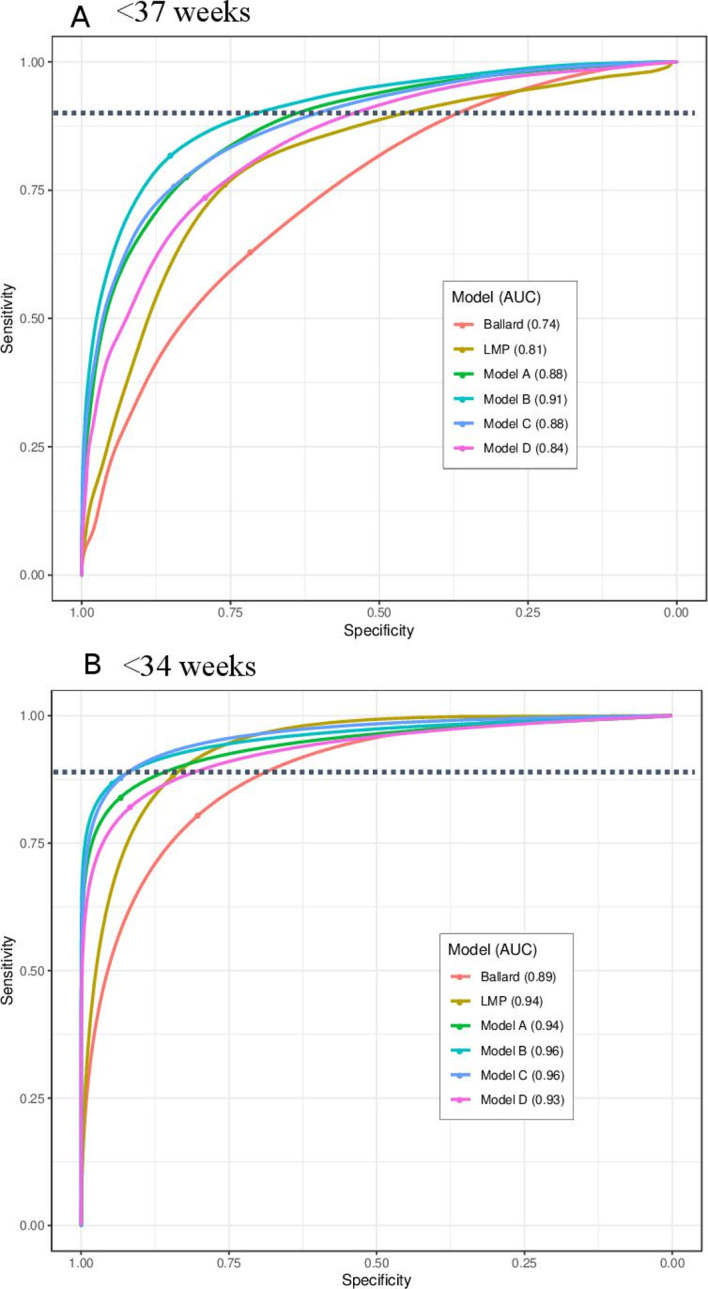
Receiver operating curves (ROC) for classification of preterm infants, (A) <37 weeks and (B) <34 weeks. Model A: 10 newborn characteristics: birth weight, head circumference, chest circumference, foot length, breast bud diameter, breast development, foot surface (plantar creases), skin texture, ankle dorsiflexion, infant sex. Model B: 10 newborn characteristics+LMP. Model C: Birth weight+LMP. Model D: Birth weight+head circumference. The point of each ROC curve intersection with the dotted line shows the point of 80% sensitivity, chosen as a desired threshold of sensitivity for a clinical screening tool. The dot on the ROC curve shows the Youden Index, the point of maximum sensitivity+specificity. AUC, area under the curve; LMP, last menstrual period.

### Machine learning models

Using machine learning, a full prediction model was built that included all 25 characteristics assessed (data not shown). Simpler models using less signs had comparable performance and are presented below. Figure 3 shows the 10 highest ranking individual predictors of GA identified during building of the machine learning models (four anthropometric measures (birth weight, head circumference, chest circumference, foot length), five physical (breast development, breast bud diameter, plantar creases on foot surface, skin texture and infant sex) and one neuromuscular (ankle dorsiflexion)).

#### Model A (10 characteristic model)

Birth weight, head circumference, chest circumference, foot length, breast bud diameter, breast development, plantar creases, skin texture, ankle dorsiflexion, infant sex: In the ‘top ten’ newborn characteristics model (model A), predicted GA values fell within ±17.3 days of early ultrasound GA (95% LOA), with an AUC of 0.88 to classify <37 week infants and 0.94 to classify <34 weeks. While the average bias was zero across all GAs, model A tended to predict higher GA compared with ultrasound dating prior to 39 weeks, and lower GA at >39 weeks (figure 2C). The sensitivity and specificity to identify preterm birth <37 weeks was 80% and 80%; and for <34 weeks 80% and 96%, respectively (table 4). The diagnostic accuracy of model A to identify preterm birth using different optimal thresholds with higher fixed sensitivity (85%, 90%, 95%) and the Youden index is shown in online supplemental table 2.

#### Model B (10 characteristic model+LMP)

LMP was additionally included with model A to determine whether the model performance and diagnostic accuracy could be further improved. In model B, the precision was further improved to 95% LOA±15.7 days (table 3). The AUCs for classification of <37 and <34 weeks were 0.91 and 0.96, respectively (table 4). At 80% sensitivity, the specificity to classify preterm births <37 weeks was 87%, and that to classify <34 week infants was 98% (alternate optimal thresholds in online supplemental table 2).

#### Model C (BW+LMP only)

In this parsimonious model with only two characteristics (model C), the 95% LOA was ±16.7 days, which was between that of model B and model A (table 3). Model C tended to predict higher GA compared with the ultrasound in the earlier GA (<37 weeks, figure 2E). At 80% sensitivity, this simplified model had 80% specificity to identify preterm <37 weeks, and 98% specificity to identify preterm <34 weeks (alternate optimal thresholds in online supplemental table 2). The AUCs for classification of <37 and <34 weeks were 0.88 and 0.96, respectively (table 4, figure 4).

#### Model D (BW+head circumference)

In this model with two best performing anthropometric measures, for use in cases when LMP may not be known (model D), 95% LOA of ±18.4 days. These LOA were marginally worse than that of models A, B and C, but substantially better than that of Ballard examination or LMP. At 80% sensitivity, model D had 72% specificity to identify preterm <37 weeks (93% specificity for <34 weeks) (alternate optimal thresholds shown in online supplemental table 2). The AUCs for classification of <37 and <34 weeks were 0.84 and 0.93, respectively (table 4, figure 4).

### Sensitivity analysis

Given that growth restriction may influence the prediction of GA based on the infant’s size, we conducted stratified analysis to explore diagnostic accuracy among infants ultimately born SGA versus AGA (appropriate for GA, >10%–90% birth weight for GA and sex) (online supplemental table 3, figure 9a–d).

Overall, the methods had more negative bias among SGA infants compared with those born AGA. Ballard examination overestimated GA on average by 10.5 days in AGA, compared with 4.9 days in SGA infants (online supplemental table 3). All machine learning models tended to systematically underestimate GA compared with ultrasound by an average of 4–5 days in SGA infants, while overestimating GA by 1 day in AGA infants. The 95% LOA were similar across models in SGA and AGA infants (online supplemental table 3). The AUCs for classification of preterm births<37 and <34 weeks were also similar for all models among SGA versus AGA infants (online supplemental table 9a–d).

## Discussion

In this large multicountry prospective pregnancy cohort with high quality early ultrasound dating (<20 weeks), we found that routine, existing methods to estimate GA, the LMP and Ballard examination, were biased and imprecise (±25–39 days). We developed a new machine learning model including 10 newborn characteristics (birth weight, head circumference, chest circumference, foot length, breast bud diameter, breast development, plantar creases, skin texture, ankle dorsiflexion, infant sex) and LMP, that estimated GA within ±15.7 days of early ultrasound dating. Furthermore, a simpler machine learning model including only birth weight and LMP performed similarly with only marginally lower diagnostic accuracy, dating 95% of pregnancies within ±16.7 days of early ultrasound.

LMP dating is still widely used for pregnancy dating in high-income countries. However, recall of LMP is less available and accurate among women of low literacy and socio-economic status.^22^ It is also subject to rounding^4–6 23 24^ (particularly to the month) and recall bias, and is less accurate for women presenting late in their antenatal care. LMP dating was biased with a significant trend, tending to underestimate GA in the lower GAs. This may have important public health implications.

The Ballard examination is one of the most common clinical methods used to estimate newborn GA after birth. In our study the Ballard was significantly biased, consistently overestimating GA by 9 days. This bias was similar across all study sites and countries. Furthermore, the estimates were imprecise, dating most pregnancies within ±25 days. These data are in line with a prior systematic review^9^ that reported that the Ballard dated 95% of pregnancies within ±3.8 weeks (26.6 days). With the low sensitivity of the Ballard examination, it correctly identified only 1 in 10 preterm infants. Widespread use of the Ballard examination for GA assessment would result in systematic overestimation of GA and under-identification of preterm infants.

In the best AMANHI machine learning model (model B), including 10 characteristics and LMP, we were able to achieve high accuracy with an AUC of 0.91, indicating that the test could correctly classify preterm or not, 91% of the time. This model predicted GA within 15.7 days of early ultrasound for 95% of newborns. Machine learning has been used by other groups and investigators to develop algorithms for estimating GA or predicting preterm birth. Rittenhouse *et al* used machine learning to develop an algorithm for predicting preterm birth in the Zambian Preterm Birth Prevention Study, with six parameters including LMP, birth weight, twin delivery, maternal height, hypertension in labour and HIV serostatus.^25^ They reported that their model correctly classified >94% of newborns as preterm or not.^25^ Torres *et al*^26^ developed a method for GA estimation using deep machine learning of newborn photos (face, foot and ear) as well as birth weight. The addition of newborn digital images improved prediction of GA by 33% compared with a model with birth weight alone.^26^

An important consideration and potential limitation of our 10 newborn characteristic model is the reliance on several measures of physical size, or anthropometric measures that reflect infant size rather than maturity. These indicators would be influenced by intrauterine growth restriction, with the exception of head circumference which is potentially less affected in cases of asymmetric intrauterine growth restriction with head sparing. The machine learning models included substantial input data from three sites with high rates of SGA (Bangladesh, Pakistan and Ghana), yet, still systematically underestimated GA among SGA infants by approximately 4–5 days. The identification and validation of novel physical or clinical characteristics to improve GA estimation among SGA and growth restricted infants is an important priority to improve GA estimation in settings where SGA prevalence is high.

Given feasibility, training and human resources considerations in LMIC, we also developed simpler two-characteristic models (models C and D), as potential alternatives to identify vulnerable babies in settings with limited resources. When LMP is known, model C (birth weight and LMP) was accurate with only marginal reductions in diagnostic accuracy compared with the best AMANHI model A. However, availability and quality of birth weight and LMP data remain major challenges in LMICs. Half of infants born in sub-Saharan Africa and Asia do not have a recorded birth weight.^27^ Poor quality of birth weight measurement is common with methodological problems including heaping of measurements, rounding, scale imprecision and lack of scale calibration. Up to one-third of women in LMICs may not recall their LMP, and accuracy of recall is less precise in lower socio-economic status and illiterate populations.^5 22^ Methods have been described to improve recall of LMP, including efforts to improve recording in ANC records, use of calendars of religious holidays or community events to prompt recall and use of menstrual calendars.^28^ In settings where LMP is not known, the two characteristic anthropometric model (model D: birth weight and head circumference) could be used instead with reasonable prediction accuracy of ±18.4 days.

There were several limitations to this study. Infants <34 weeks infants represented only 1% of this analysed cohort. About 28% of infants <34 weeks died before the assessment. Given the exclusion of critically ill infants, the effect of morbidities on model performance cannot be assessed. Another consideration is the timing of the assessment. Certain physical signs may vary after birth, such as the skin opacity or foot creases. In our study, the majority of assessments (80%) were conducted within 48 hours of birth. We had hypothesised that feeding maturity observations and signs may improve performance in estimating gestational maturity. However, we found feeding questions difficult to train and standardise across sites, and these variables dropped out of the modelling in early stages.

## Conclusion

The WHO AMANHI machine learning GA model including 10 newborn characteristics and LMP predicted GA within ±16 days of an early pregnancy ultrasound, and a simpler machine learning model including only birth weight and LMP performed well with modest reductions in prediction accuracy. This accuracy is similar to the traditional clinical 21-sign Dubowitz examination and substantially more accurate and less biased than the Ballard examination. These new machine learning models hold promise for accurate and timely identification of vulnerable, preterm infants -- the essential first step required in order to provide them with the special care needed to reduce global neonatal morbidity and mortality.

## Data Availability

Data are available upon reasonable request. Please note that data will be made available upon the agreement of all PIs and WHO, exclusively for non-commercial purposes. All requests related to data sharing should be sent to Dr Rajiv Bahl (bahlr@who.int).
