# Supplementary material for: Simplified models to assess newborn gestational age in low-middle income countries: findings from a multicountry, prospective cohort study
Source: BMJ Glob Health. 2021 Sep 13;6(9):e005688. doi: 10.1136/bmjgh-2021-005688 (PMC8438948; doi:10.1136/bmjgh-2021-005688)
Supplement: Supplementary data [file bmjgh-2021-005688supp001.pdf]

WEBAPPENDIX: WHO AMANHI Gestational Age Study

Machine Learning Models to Assess Newborn Gestational Age in Low-Middle Income Countries: Findings from a multi-country, prospective cohort study

Table of Contents

Web Table 1: Detailed description of the Study Sites and Procedures 2

Web Table 2. Diagnostic accuracy of Ballard, LMP, and AMANHI models for identification of newborns <37 and <34 weeks 3

Web Table 3. Stratified analysis by SGA vs AGA Status 5

Web Figure 2a-f. Bangladesh Bland Altman Curves 5

Web Figure 3a-f. Ghana Bland Altman Curves 7

Web Figure 4a-f. Pakistan (Karachi) Bland Altman Curves 8

Web Figure 5a-f. Tanzania (Pemba) Bland Altman Curves 10

Web Figure 6a-f. Zambia Bland Altman Curves 11

Web Figure 7a-e. Receiver Operating Curves for the identification of newborns <37 weeks 12

Web Figure 8.a-e Receiver Operating Curves for the identification of newborns <34 weeks 12

Web Figure 9a-d. ROC Curves by AGA and SGA for classification of newborns <37 weeks and <34 weeks 12

. *BMJ Global Health* 2021; 6:e005688. doi: 10.1136/bmjgh-2021-005688

Web Table 1: Detailed description of the Study Sites and Procedures

	Bangladesh	Ghana	Pakistan (Karachi)	Tanzania (Pemba)	Zambia
**Site Location/ Description**	Rural areas of Sylhet district	Rural areas in central Ghana	1 peri-urban community of Bin Qasim town and 1 peri-urban community of Korangi town, Karachi	Pemba Island in the Zanzibar archipelago	6 rural districts in Southern Province
**Pregnancy surveillance**	2-monthly by trained community health workers (CHWs)	Monthly fieldworkers (FW)	3-monthly by FWs	6-weekly by CHWs	Facility ANC enrollment
**Population area[1]**	88,000	147,000	97000	72,000	25,000
**Ultrasonography**	Fujifilm Sonosite M-Turbo® ultrasound system (Bothel, WA, US). Scans performed by trained MD sonographers.	Portable SonoSite™ S180 machines (SonoSite™, Inc, Bothel, WA, US). Scan performed by hospital midwives.	Mindray 7 Ultrasound System. Scan done by trained sonologist.	Sonoscape s6. Scans performed by trained sonographer in district hospitals.	Philips Sono Diagnost 260; Scans performed by trained sonographers.
**% Facility Delivery[1]**	43.7	79.9	64.3	99 (?)	97.1
**% with newborn visit completed <72 hours**	71.1%	99.2%	80.0%	92.7%	82.6%
**Infant Weighing Scale**	TANITA BD-585 Pediatric Scale Digital weighing scale. Precision 10 gm.	Salter suspension scale (non-digital) with sling. Precision 100 gm.	LAICA Electronic/digital infant scale. Precision 5 gm.	Seca 374 (capacity 44lb Digital infant weighing scale Precision 10 gm	GPC Gps092 Medical hanging scale (Delhi, India). Precision 100 grams.
**Health worker type performing newborn assessment**	Locally recruited women (non-clinical) with at least 10 grade education received 6 weeks training on maternal and newborn health; additional 7 days training on newborn assessment	Locally recruited non-clinical field supervisors with secondary school education. Trained for 7 days on newborn assessment.	Locally recruited women with at least 12 grade education received 6 month training on maternal and newborn care; additional 7 days training on newborn assessment	Trained health worker (health assistant) with 2 years formal health training. Initial 7 days training for newborn assessment.	Locally recruited women (non-clinical) with 12 years of schooling; Trained for 3 days on newborn assessment.
**% SGA[1]**	41.8	33.4	35.5	9.6	17.8
**% LBW[1]**	25.6	11.8	22.5	4.6	6.5
**NMR[1]**	37.8 (35.5-40.2)	29.1 (26.9-31.2)	50.1 (46.9-53.4)	16.0 (14.3-17.8)	14·5 (13·0–16·0)

[1]Population-based rates, timing, and causes of maternal deaths, stillbirths, and neonatal deaths in south Asia and sub-Saharan Africa: a multi-country prospective cohort study. Lancet Glob Health 2018; 6(12): e1297-e308.

. *BMJ Global Health* 2021; 6:e005688. doi: 10.1136/bmjgh-2021-005688

Web Table 2. Diagnostic accuracy of Ballard, LMP, and AMANHI models for identification of <37 week and <34 week infants

Gold standard dated by early pregnancy ultrasound, additional clinical thresholds maximizing sensitivity for identification of preterm births.

Model	AUC	Threshold selection	Se	Sp	PPV	NPV	LRP	LRN
**Classify <37 weeks**								
LMP	0.81	Youden Index	0.76	0.76	0.20	0.96	2.38	0.51
LMP	0.81	95% sensitivity	0.95	0.23	0.09	0.98	1.23	0.22
LMP	0.81	90% sensitivity	0.90	0.36	0.11	0.98	1.66	0.22
LMP	0.81	85% sensitivity	0.85	0.61	0.14	0.98	2.17	0.25
Ballard exam	0.74	Youden Index	0.63	0.71	0.15	0.96	2.20	0.52
Ballard	0.74	95% sensitivity	0.95	0.25	0.09	0.98	1.26	0.20
Ballard	0.74	90% sensitivity	0.90	0.37	0.10	0.98	1.42	0.27
Ballard	0.74	85% sensitivity	0.85	0.45	0.11	0.97	1.55	0.33
Model A (10-characteristics)	0.88	Youden Index	0.78	0.82	0.25	0.98	4.35	0.27
Model A	0.88	95% sensitivity	0.95	0.45	0.12	0.99	1.74	0.11
Model A	0.88	90% sensitivity	0.90	0.64	0.16	0.99	2.48	0.16
Model A	0.88	85% sensitivity	0.85	0.73	0.20	0.98	3.15	0.21
Model B (10-characteristics+LMP)	0.91	Youden Index	0.82	0.85	0.30	0.98	5.40	0.21
Model B	0.91	95% sensitivity	0.95	0.52	0.13	0.99	1.96	0.10
Model B	0.91	90% sensitivity	0.90	0.71	0.19	0.99	3.07	0.14
Model B	0.91	85% sensitivity	0.85	0.81	0.26	0.99	4.49	0.19
Model C (BW+LMP)	0.88	Youden Index	0.76	0.84	0.27	0.98	4.83	0.29
Model C	0.88	95% sensitivity	0.95	0.42	0.11	0.99	1.65	0.12
Model C	0.88	90% sensitivity	0.90	0.60	0.15	0.99	2.27	0.17
Model C	0.88	85% sensitivity	0.85	0.72	0.19	0.98	3.03	0.21
Model D (BW+HC)	0.84	Youden Index	0.74	0.79	0.22	0.97	3.53	0.33
Model D	0.84	95% sensitivity	0.95	0.38	0.11	0.99	1.54	0.13
Model D	0.84	90% sensitivity	0.90	0.54	0.13	0.99	1.97	0.18
Model D	0.84	85% sensitivity	0.85	0.65	0.16	0.98	2.40	0.23
**Classify <34 weeks**								
LMP	0.94	Youden Index	0.88	0.84	0.05	1.00	5.58	0.14
LMP	0.94	95% sensitivity	0.95	0.74	0.03	1.00	3.65	0.07
LMP	0.94	90% sensitivity	0.90	0.82	0.04	1.00	5.13	0.12
LMP	0.94	85% sensitivity	0.85	0.87	0.06	1.00	6.57	0.17
Ballard	0.89	Youden Index	0.81	0.80	0.03	1.00	4.04	0.24
Ballard	0.89	95% sensitivity	0.95	0.53	0.02	1.00	2.02	0.09
Ballard	0.89	90% sensitivity	0.90	0.67	0.02	1.00	2.70	0.15
Ballard	0.89	85% sensitivity	0.85	0.75	0.03	1.00	3.39	0.20
Model A (10-characteristics)	0.94	Youden Index	0.84	0.93	0.10	1.00	12.28	0.17
Model A	0.94	95% sensitivity	0.95	0.62	0.02	1.00	2.47	0.08
Model A	0.94	90% sensitivity	0.90	0.83	0.05	1.00	5.39	0.12
Model A	0.94	85% sensitivity	0.85	0.92	0.09	1.00	10.93	0.16
Model B (10-characteristics+LMP)	0.96	Youden Index	0.87	0.95	0.12	1.00	15.91	0.14
Model B	0.96	95% sensitivity	0.95	0.72	0.03	1.00	3.38	0.07
Model B	0.96	90% sensitivity	0.90	0.90	0.08	1.00	9.11	0.11
Model B	0.96	85% sensitivity	0.85	0.96	0.16	1.00	22.01	0.16
Model C (BW+LMP)	0.96	Youden Index	0.88	0.93	0.10	1.00	12.88	0.13
Model C	0.96	95% sensitivity	0.95	0.78	0.04	1.00	4.25	0.06
Model C	0.96	90% sensitivity	0.90	0.91	0.08	1.00	9.56	0.11
Model C	0.96	85% sensitivity	0.85	0.95	0.14	1.00	18.71	0.16
Model D (BW+HC)	0.93	Youden Index	0.82	0.92	0.08	1.00	9.72	0.19
Model D	0.93	95% sensitivity	0.95	0.56	0.02	1.00	2.18	0.09

Model D	0.93	90% sensitivity	0.90	0.78	0.04	1.00	4.10	0.13
Model D	0.93	85% sensitivity	0.85	0.88	0.06	1.00	7.19	0.17

. *BMJ Global Health* 2021; 6:e005688. doi: 10.1136/bmjgh-2021-005688

Web Table 3. Stratified analysis by SGA vs AGA Status

	SGA			AGA		
**Model**	**Mean Difference**	**Bland Altman Limits of Agreement**	**BA LOA**	**Mean Difference**	**Bland Altman Limits of Agreement**	**BA LOA**
**LMP**	-0.64 (-1.52, -0.25)	(-40.6, 39.3)	40.0	-1.42 (-2.54, -1.98)	(-41.2, 37.3)	39.2
**Ballard**	4.85 (4.31, 5.39)	(-19.7, 29.4)	24.6	10.53 (10.19, 10.86)	(-13.0, 34.1)	23.5
**Model A (10 characteristics)**	-5.05 (-5.38, -4.71)	(-20.1, 10.0)	15.0	1.66 (1.43, 1.88)	(-14.4, 17.7)	16.0
**Model B (10 characteristics+LMP)**	-4.03 (-4.35, -3.72)	(-18.2, 10.1)	14.2	1.31 (1.10, 1.52)	(-13.3, 15.9)	14.6
**Model C (BW+LMP)**	-4.49 (-4.83, -4.15)	(-19.8, 10.9)	15.4	1.50 (1.29, 1.72)	(-13.8, 16.8)	15.3
**Model D (BW+HC)**	-5.61 (-5.95, -5.27)	(-20.9, 9.7)	15.3	1.93 (1.68, 2.17)	(-15.1, 19.0)	17.0

Web Figure 2a-f. Bangladesh Bland Altman Curves

2a. Bangladesh LMP vs Ultrasound

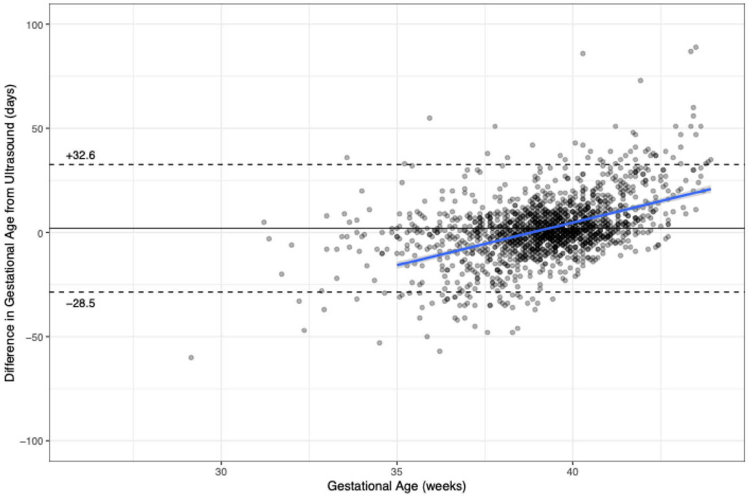

2b. Bangladesh Ballard vs Ultrasound

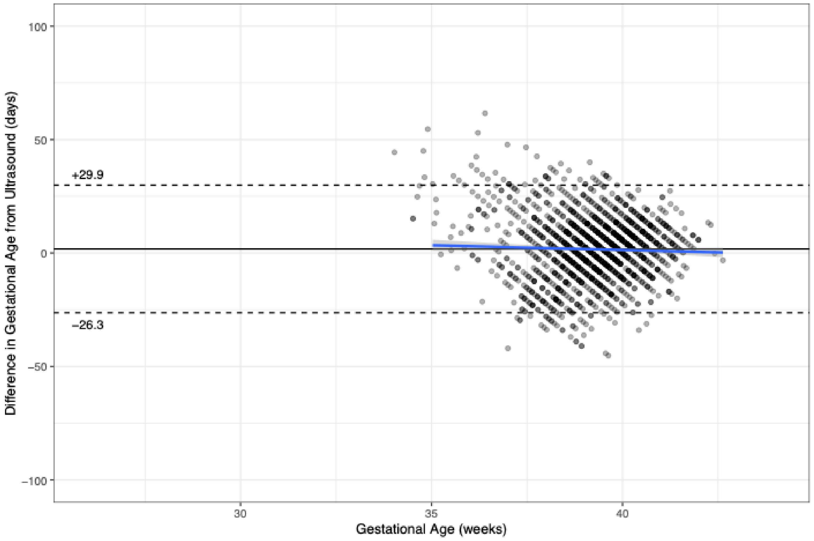

. *BMJ Global Health* 2021; 6:e005688. doi: 10.1136/bmjgh-2021-005688

2c. Bangladesh Model A vs Ultrasound

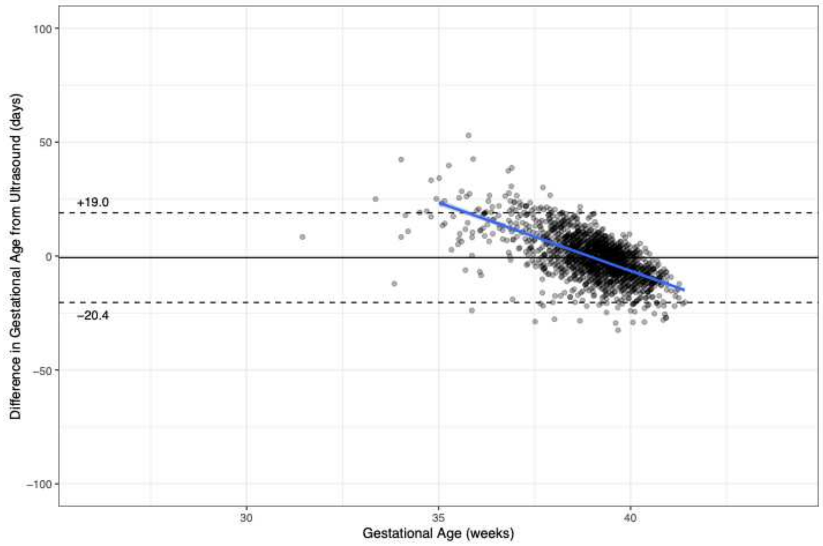

2d. Bangladesh Model B vs Ultrasound

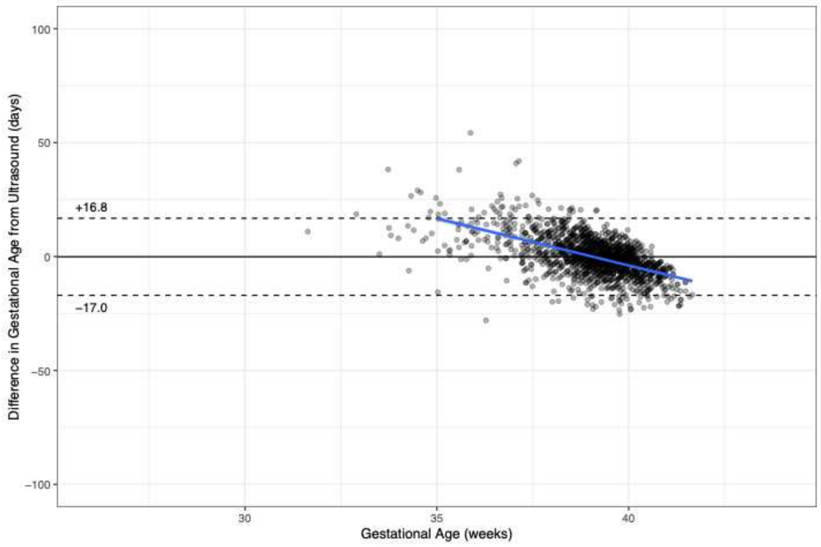

2e. Bangladesh Model C vs Ultrasound

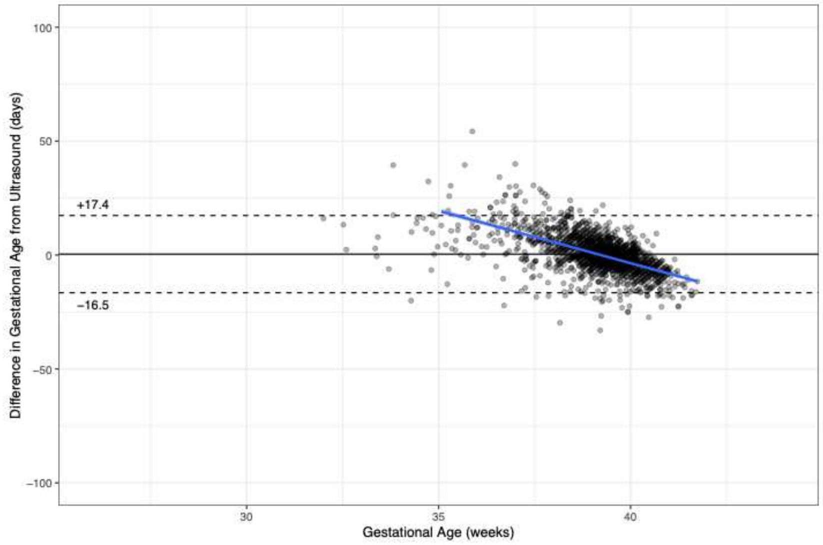

2f. Bangladesh Model D vs Ultrasound

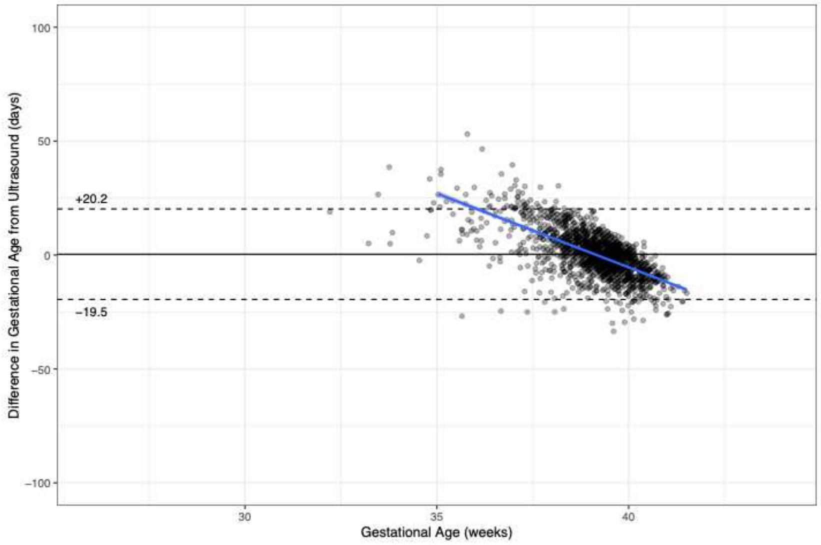

. *BMJ Global Health* 2021; 6:e005688. doi: 10.1136/bmjgh-2021-005688

Web Figure 3a-f. Ghana Bland Altman Curves

3a. Ghana LMP vs Ultrasound

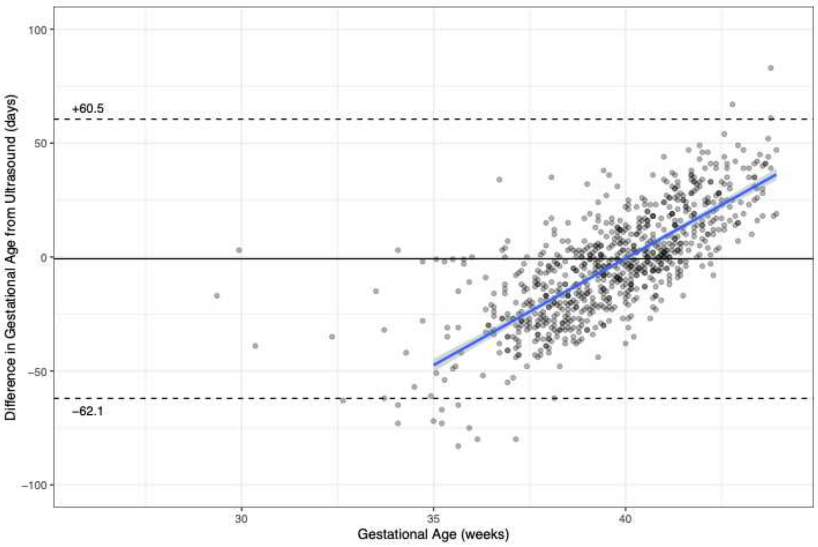

3b. Ghana Ballard vs Ultrasound

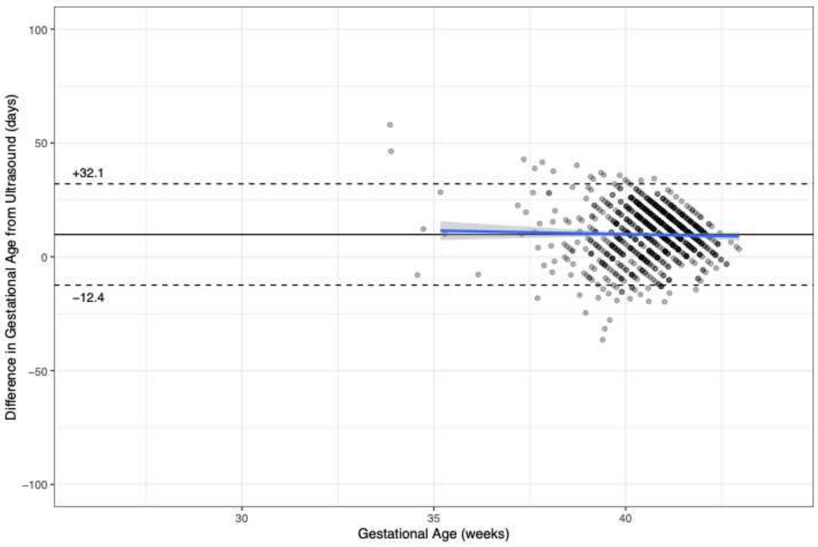

3c. Ghana Model A vs Ultrasound

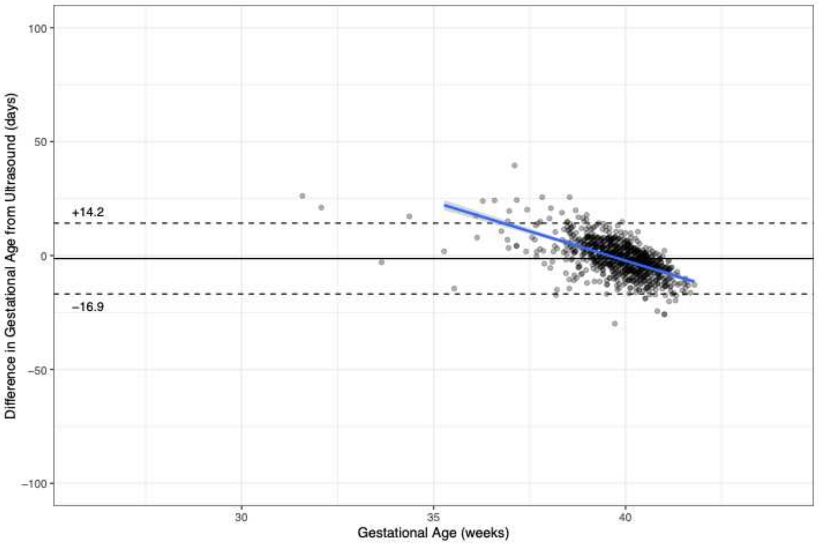

3d. Ghana Model B vs Ultrasound

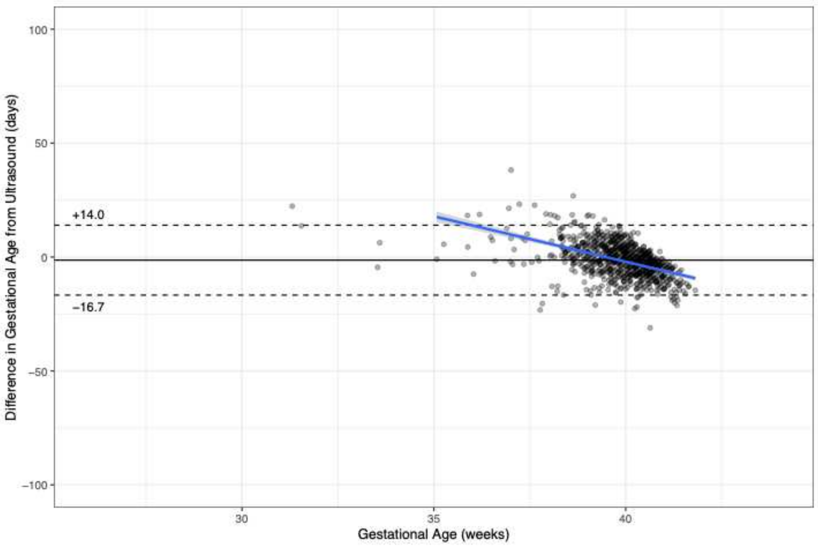

. *BMJ Global Health* 2021; 6:e005688. doi: 10.1136/bmjgh-2021-005688

3e. Ghana Model C vs Ultrasound

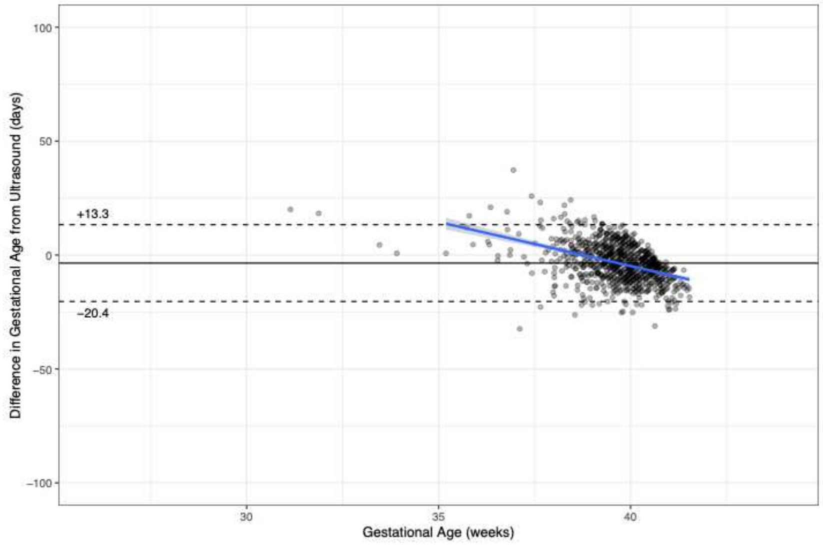

3f. Ghana Model D vs Ultrasound

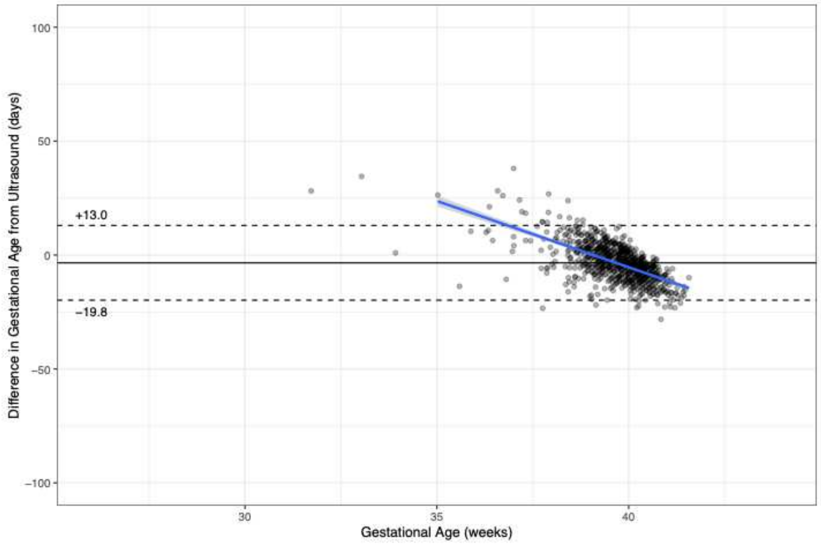

Web Figure 4a-f. Pakistan (Karachi) Bland Altman Curves

4a. Karachi LMP vs Ultrasound

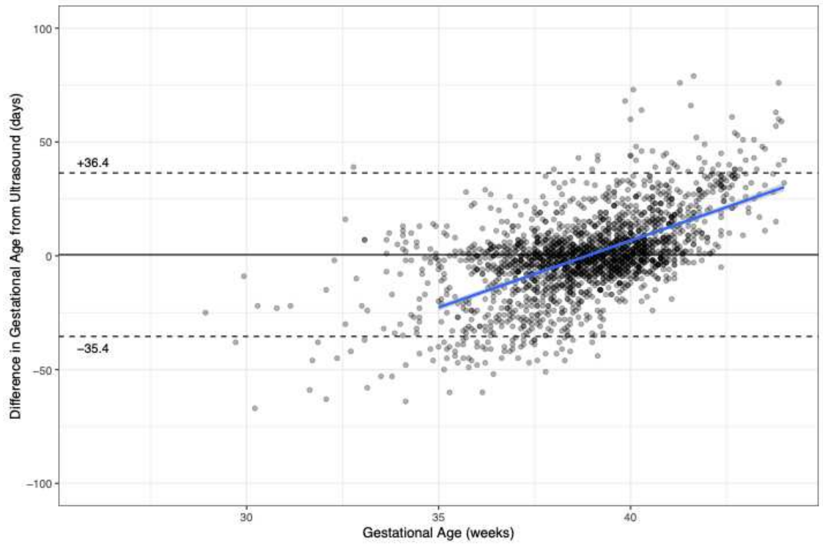

4b. Karachi Ballard vs Ultrasound

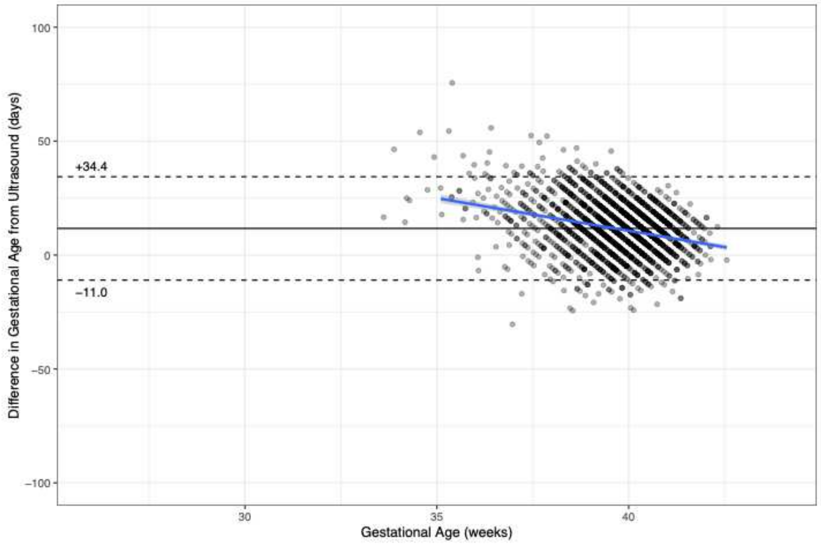

4c. Karachi Model A vs Ultrasound

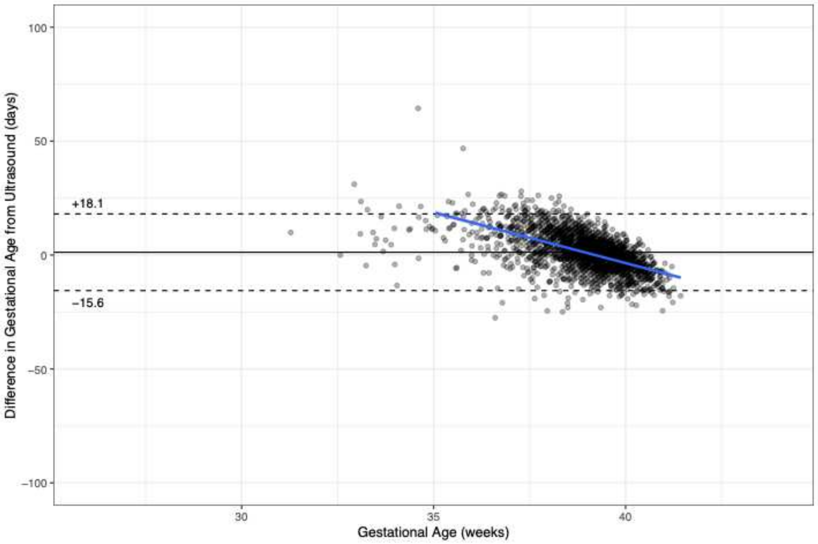

4d. Karachi Model B vs Ultrasound

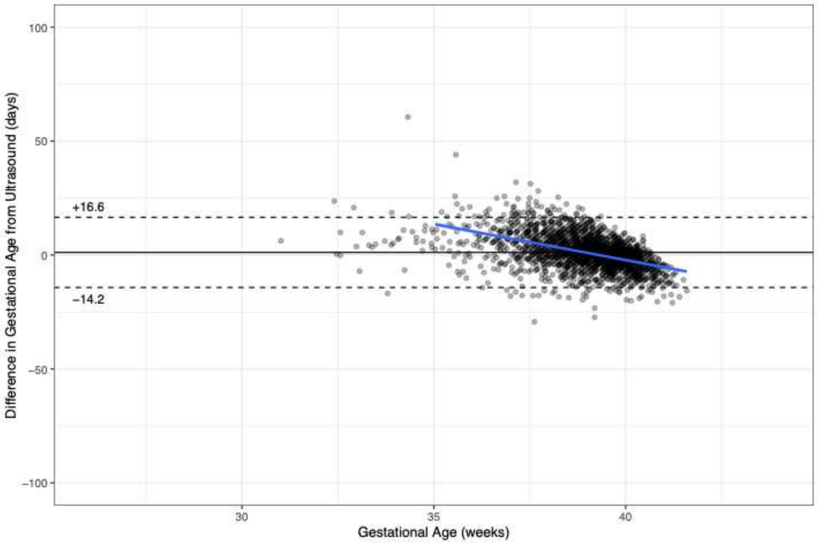

4e. Karachi Model C vs Ultrasound

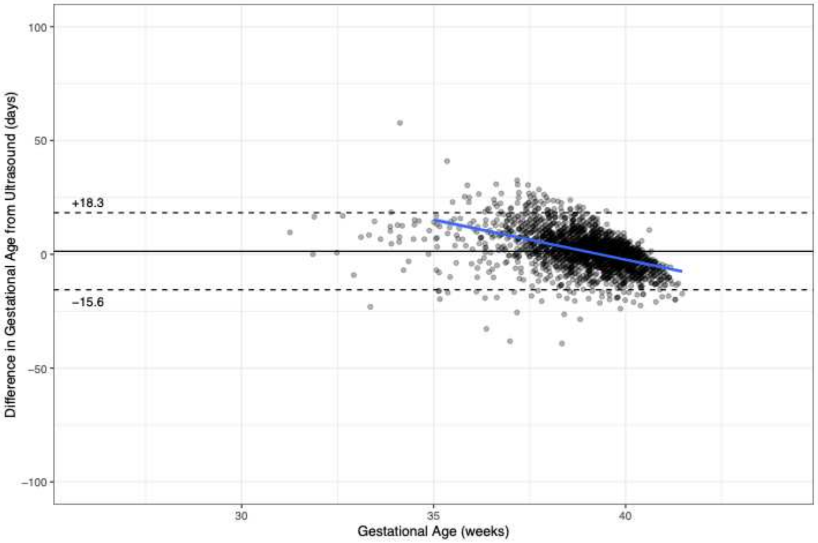

4f. Karachi Model D vs Ultrasound

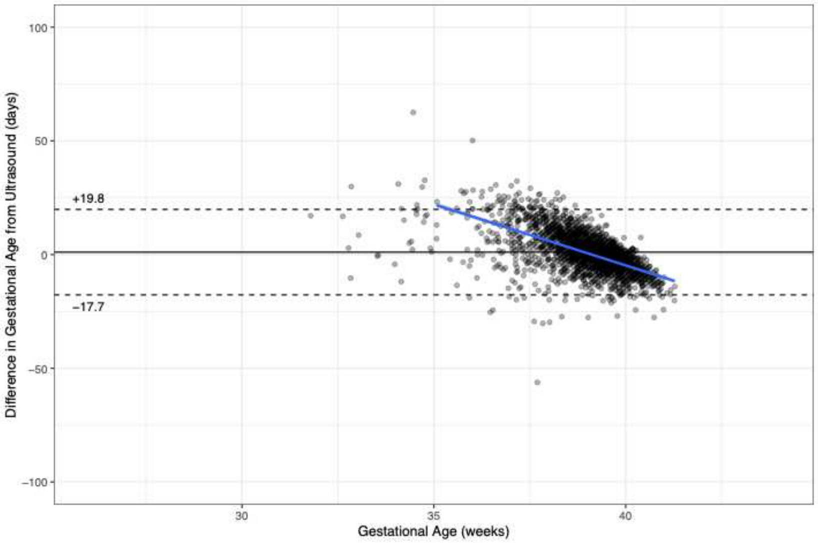

. *BMJ Global Health* 2021; 6:e005688. doi: 10.1136/bmjgh-2021-005688

Web Figure 5a-f. Tanzania (Pemba) Bland Altman Curves

5a. Pemba LMP vs Ultrasound

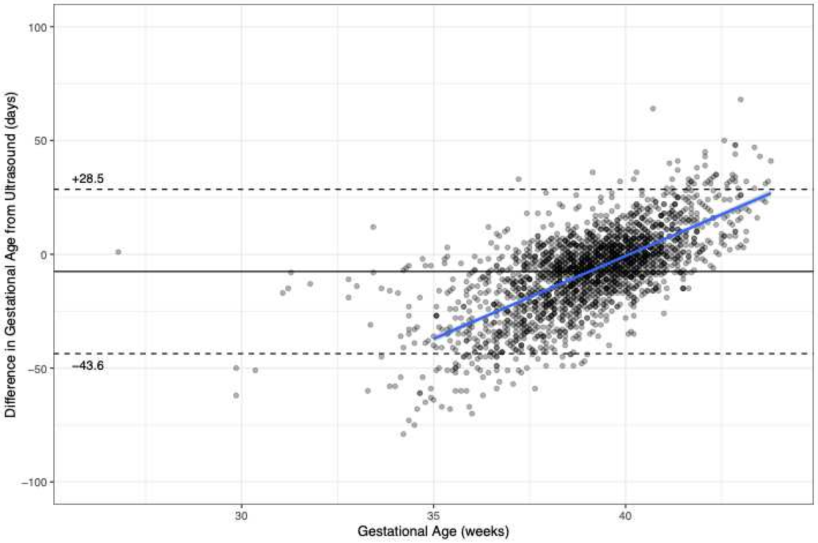

5b. Pemba Ballard vs Ultrasound

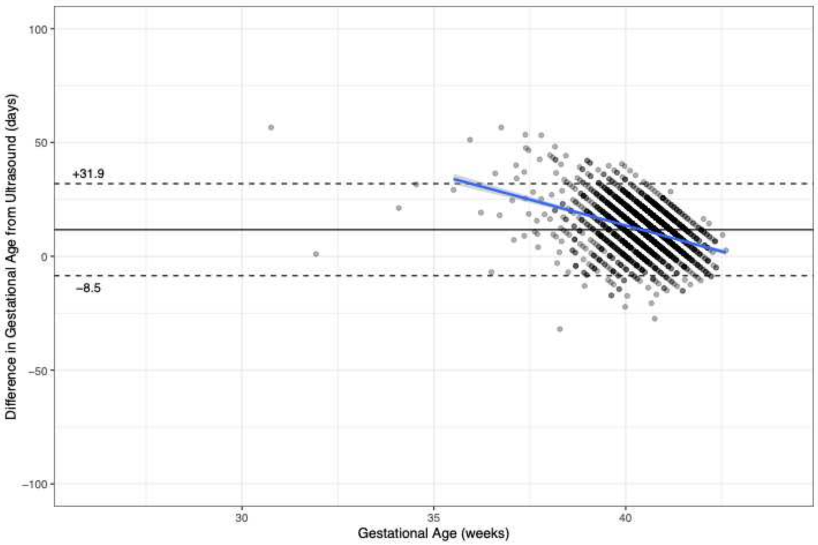

5c. Pemba Model A vs Ultrasound

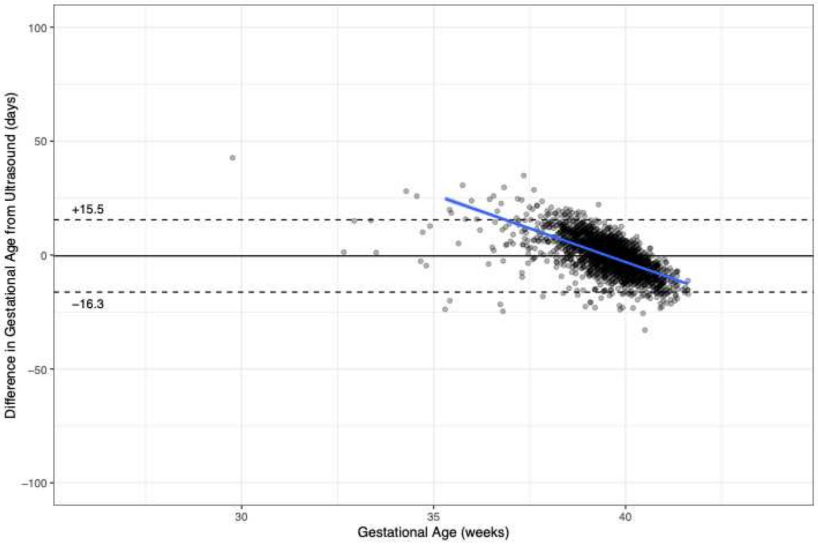

5d. Pemba Model B vs Ultrasound

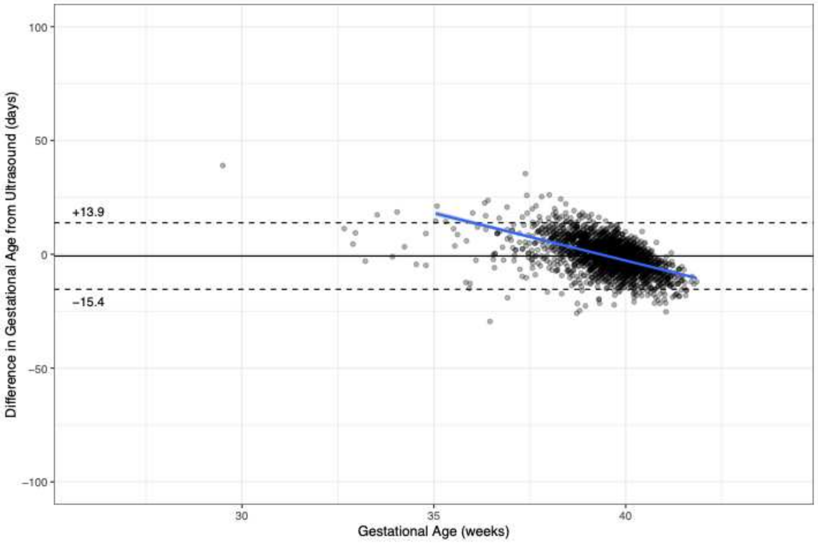

. *BMJ Global Health* 2021; 6:e005688. doi: 10.1136/bmjgh-2021-005688

5e. Pemba Model C vs Ultrasound

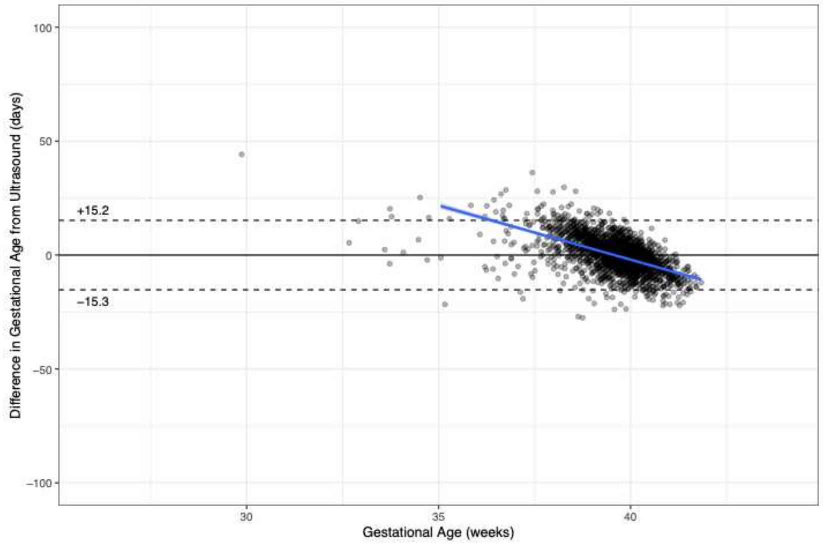

5f. Pemba Model D vs Ultrasound

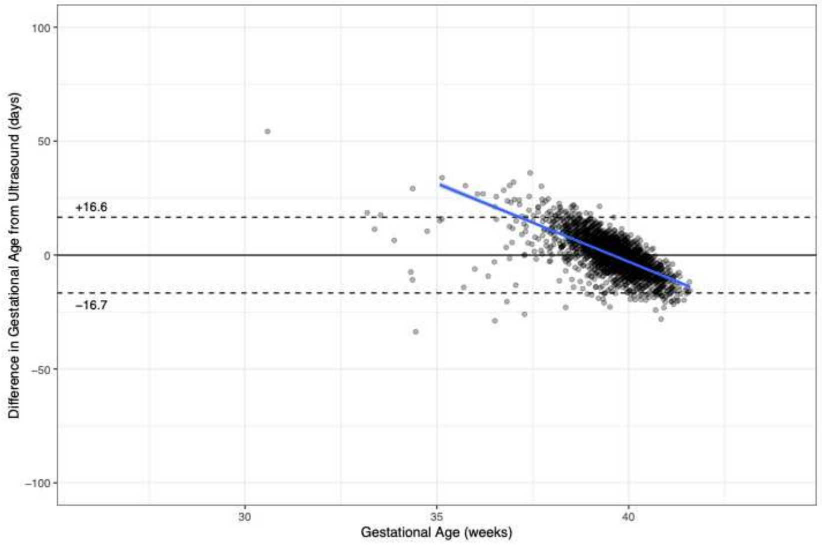

Web Figure 6a-f. Zambia Bland Altman Curves

6a. Zambia LMP vs Ultrasound

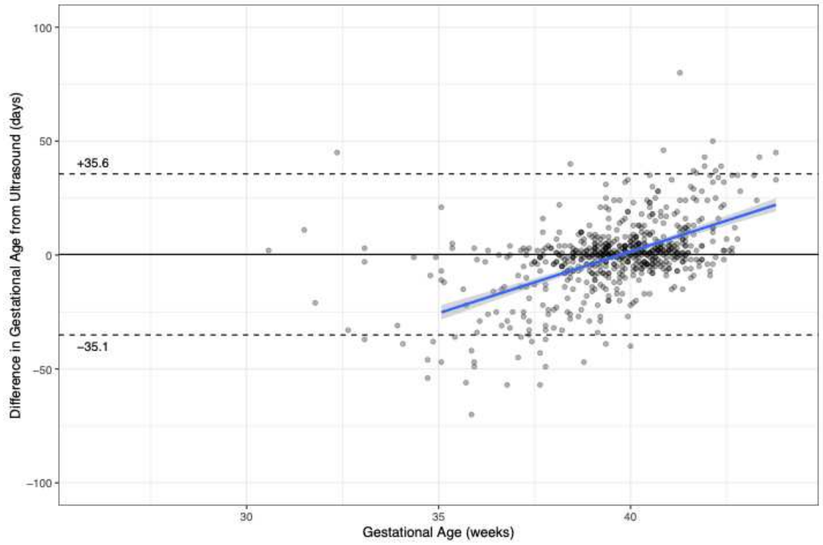

6b. Zambia Ballard vs Ultrasound

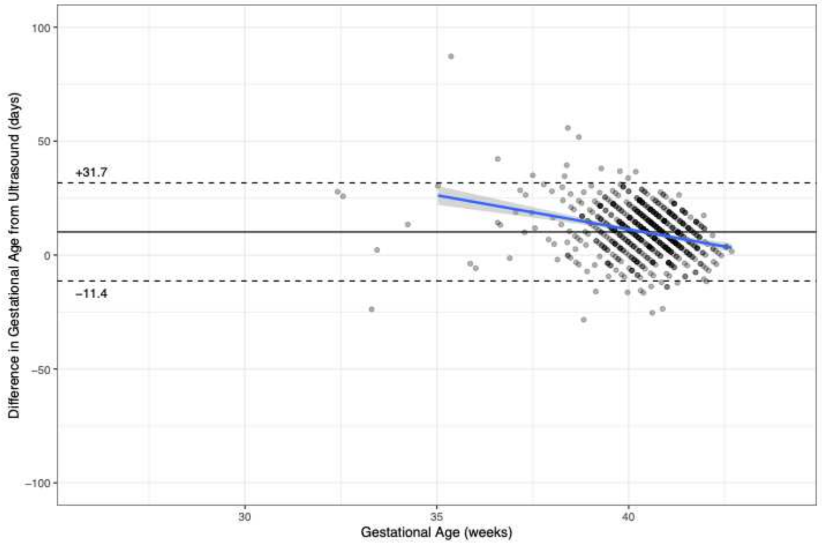

6c. Zambia Model A vs Ultrasound

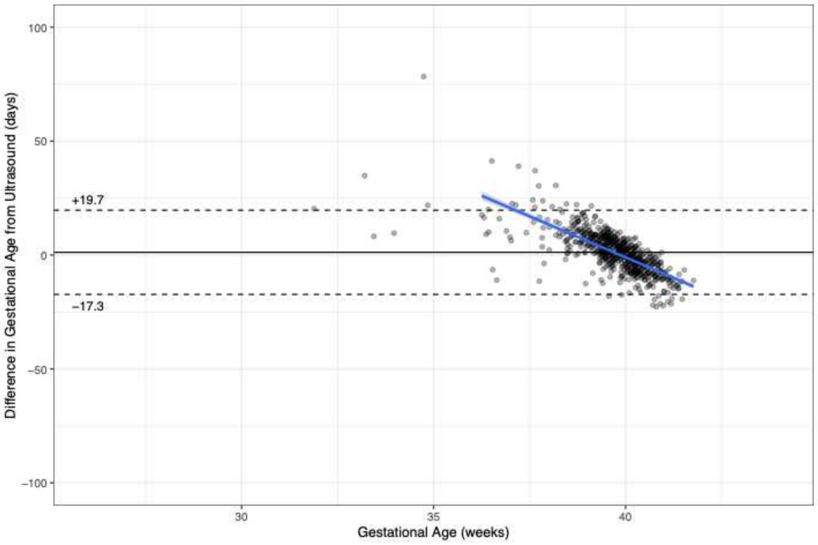

6d. Zambia Model B vs Ultrasound

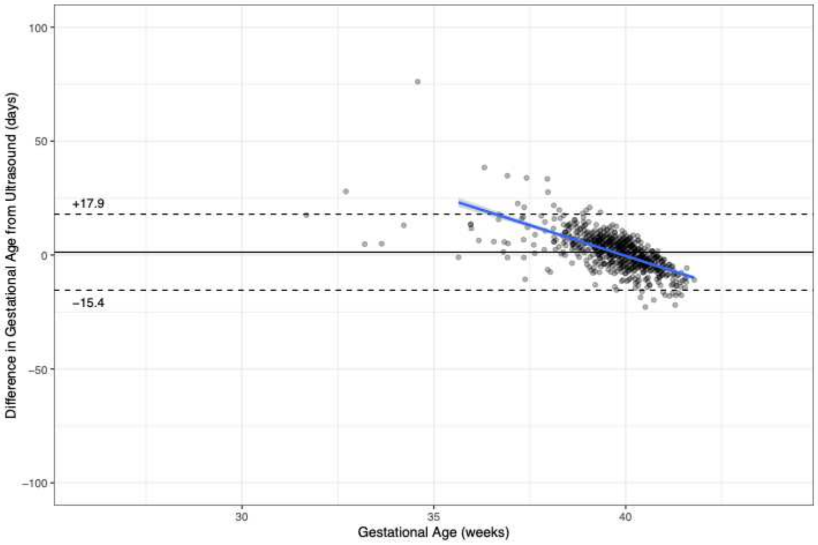

6e. Zambia Model C vs Ultrasound

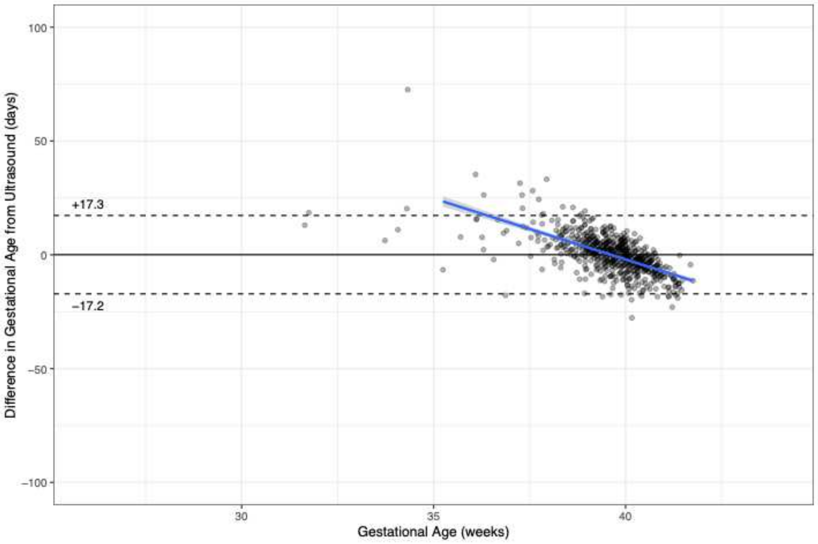

6f. Zambia Model D vs Ultrasound

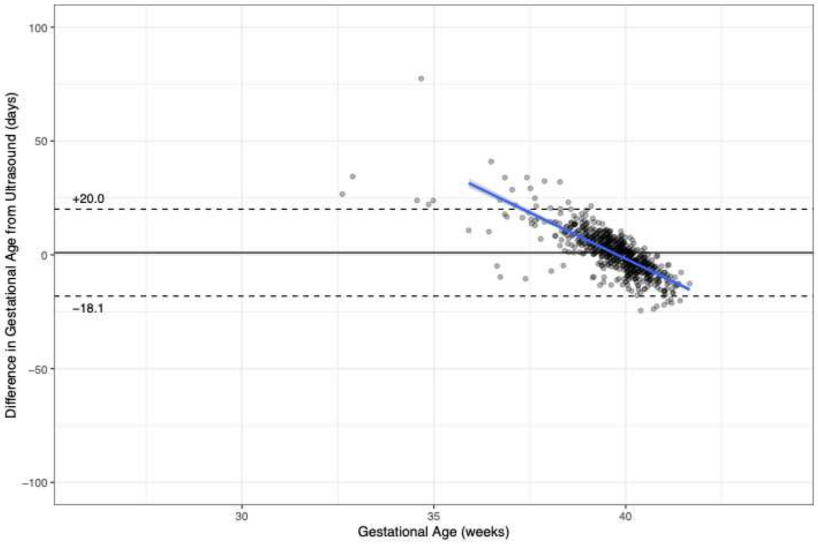

. *BMJ Global Health* 2021; 6:e005688. doi: 10.1136/bmjgh-2021-005688

Web Figure 7a-e. Receiver Operating Curves for the Identification of Preterm Births (<37 weeks)

WHO AMANHI Cohorts - Site Specific Data

7a. Bangladesh

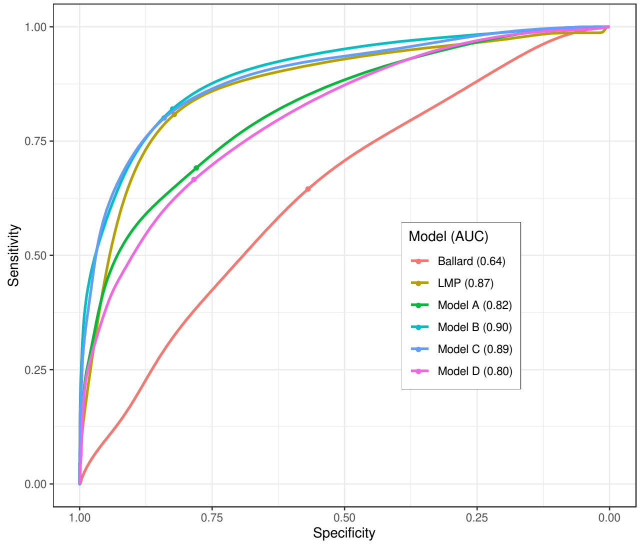

7b. Ghana

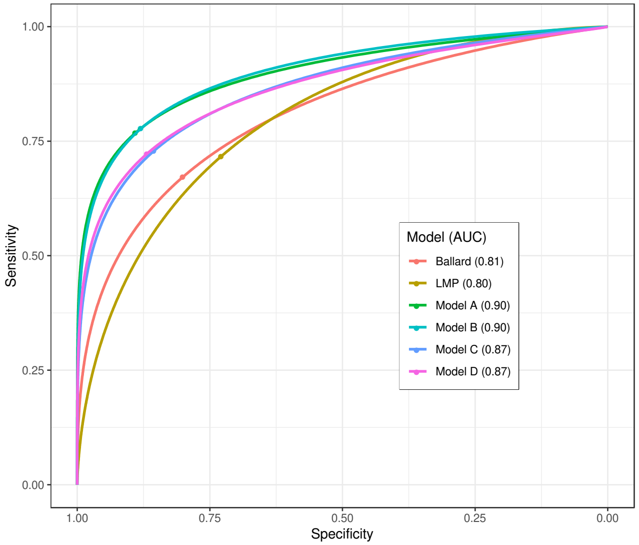

7c. Pakistan (Karachi)

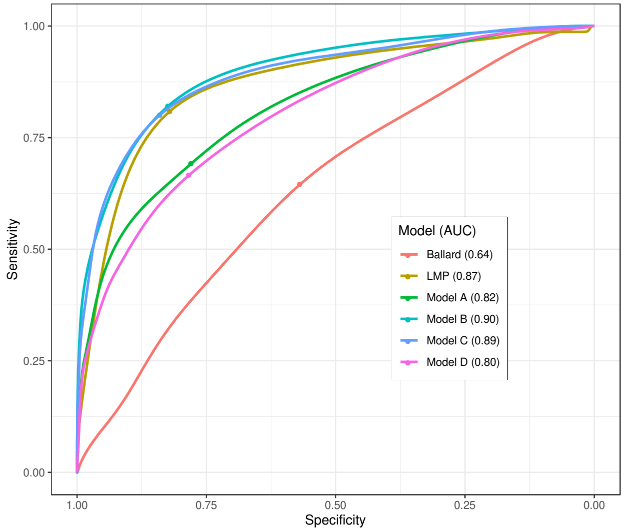

7d. Tanzania (Pemba)

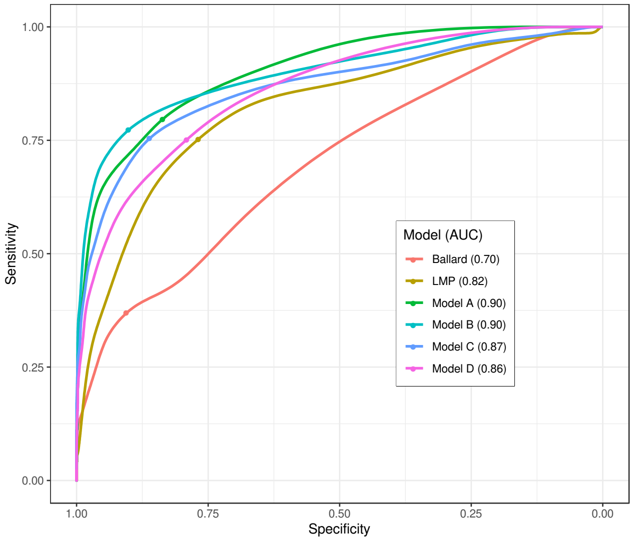

. *BMJ Global Health* 2021; 6:e005688. doi: 10.1136/bmjgh-2021-005688

7e. Zambia

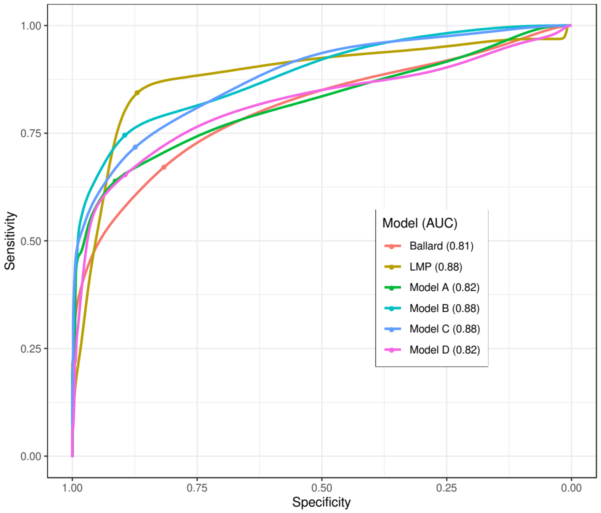

Web Figure 8.a-e Receiver Operating Curves for the Identification of Preterm Births (<34 weeks)

WHO AMANHI Cohorts - Site Specific Data

8a. Bangladesh

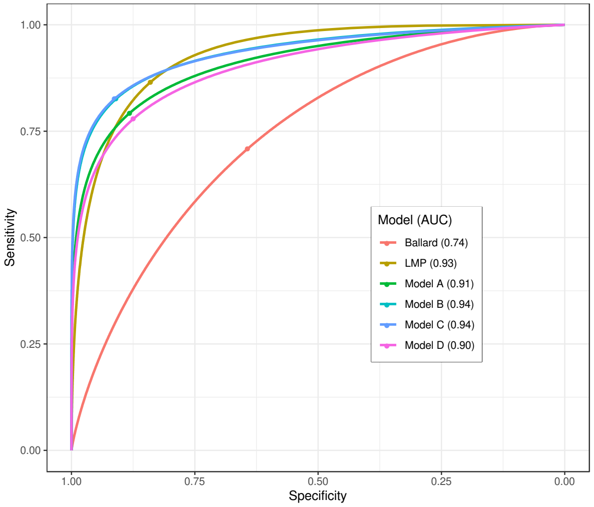

8b. Ghana

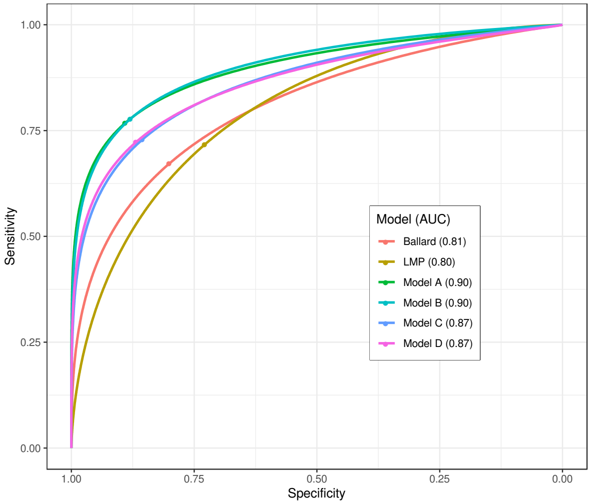

8c. Pakistan (Karachi)

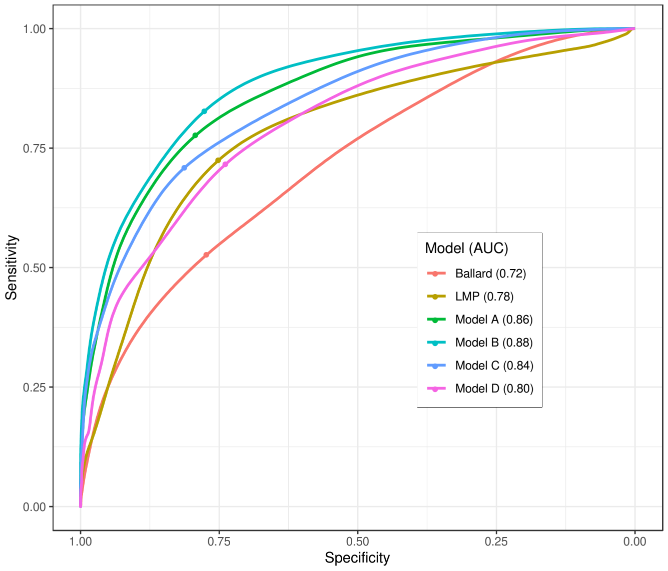

8d. Tanzania (Pemba)

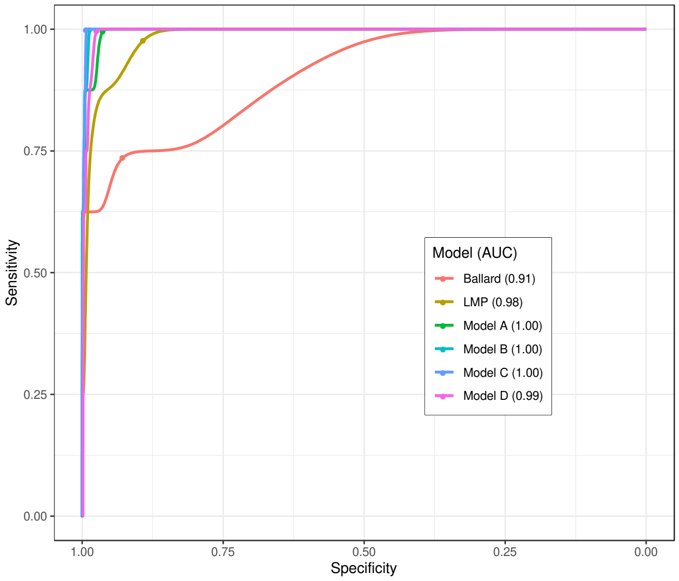

8e. Zambia

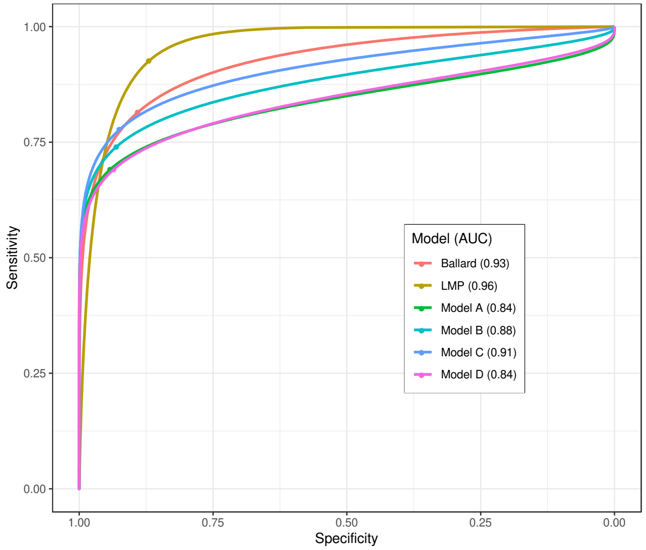

. *BMJ Global Health* 2021; 6:e005688. doi: 10.1136/bmjgh-2021-005688

Web Figure 9a-d. ROC Curves by AGA and SGA status for classification of infants <37 weeks and <34 weeks

9a. Preterm (<37 weeks) classification among AGA infants

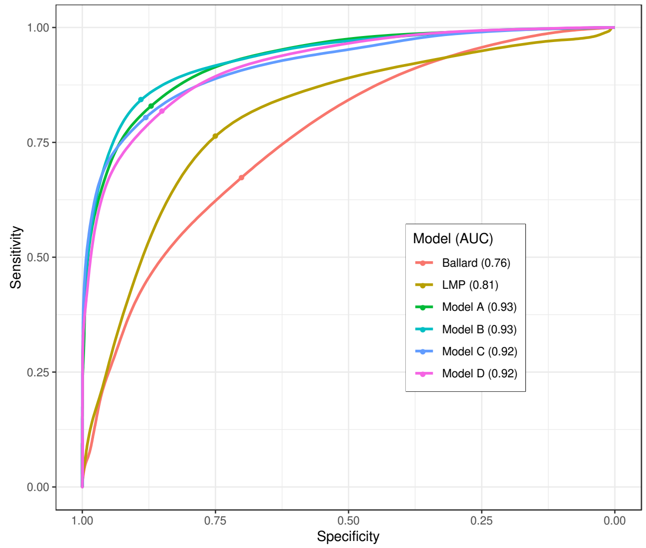

9b. Preterm (<37 weeks) classification among SGA infants

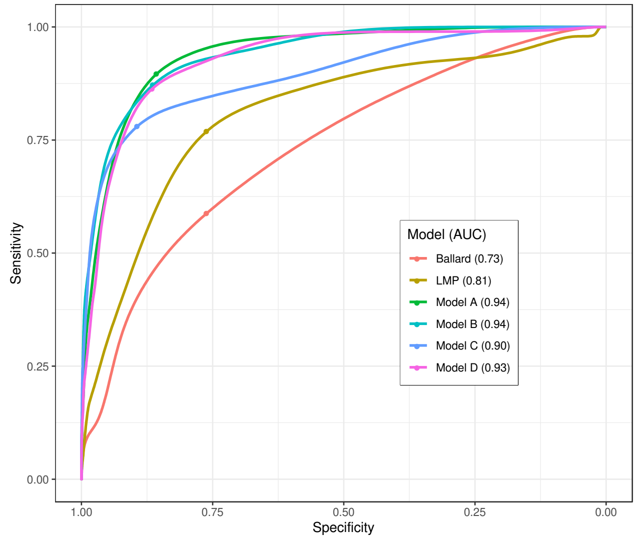

9c. Early preterm (<34 wk) classification among AGA infants

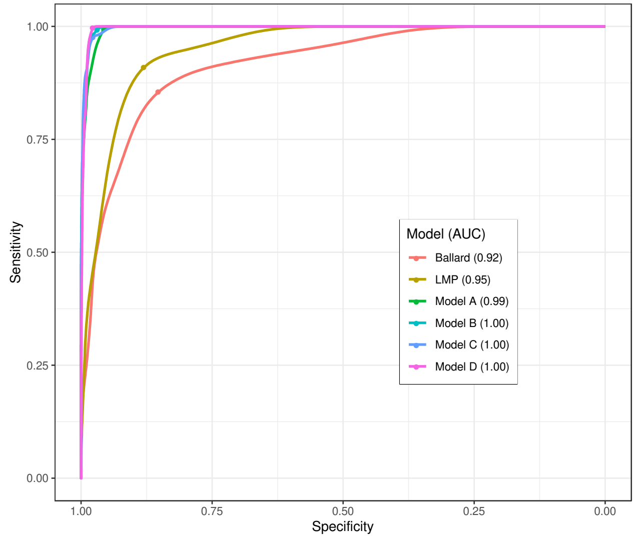

9d. Early preterm (<34 wk) classification among SGA infants

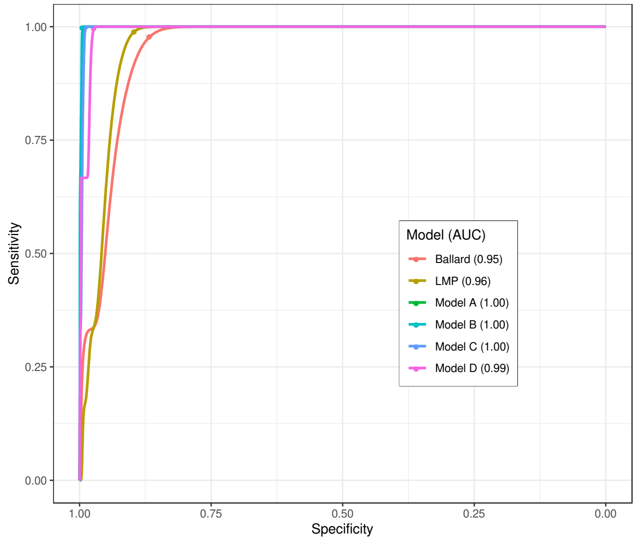

. *BMJ Global Health* 2021; 6:e005688. doi: 10.1136/bmjgh-2021-005688
